# Macrophages in cardiovascular diseases: molecular mechanisms and therapeutic targets

**DOI:** 10.1038/s41392-024-01840-1

**Published:** 2024-05-31

**Authors:** Runkai Chen, Hongrui Zhang, Botao Tang, Yukun Luo, Yufei Yang, Xin Zhong, Sifei Chen, Xinjie Xu, Shengkang Huang, Canzhao Liu

**Affiliations:** 1 Department of Cardiology, Laboratory of Heart Center, Heart Center, Translational Medicine Research Center, Zhujiang Hospital, Southern Medical University, 253 Industrial Avenue, Guangzhou, 510280 China; 2 State Key Laboratory of Cardiovascular Disease, Fuwai Hospital, National Center for Cardiovascular Diseases, Chinese Academy of Medical Sciences and Peking Union Medical College, Beijing, 100037 China

**Keywords:** Cardiology, Cell biology, Molecular biology, Cardiovascular diseases, Inflammation

## Abstract

The immune response holds a pivotal role in cardiovascular disease development. As multifunctional cells of the innate immune system, macrophages play an essential role in initial inflammatory response that occurs following cardiovascular injury, thereby inducing subsequent damage while also facilitating recovery. Meanwhile, the diverse phenotypes and phenotypic alterations of macrophages strongly associate with distinct types and severity of cardiovascular diseases, including coronary heart disease, valvular disease, myocarditis, cardiomyopathy, heart failure, atherosclerosis and aneurysm, which underscores the importance of investigating macrophage regulatory mechanisms within the context of specific diseases. Besides, recent strides in single-cell sequencing technologies have revealed macrophage heterogeneity, cell–cell interactions, and downstream mechanisms of therapeutic targets at a higher resolution, which brings new perspectives into macrophage-mediated mechanisms and potential therapeutic targets in cardiovascular diseases. Remarkably, myocardial fibrosis, a prevalent characteristic in most cardiac diseases, remains a formidable clinical challenge, necessitating a profound investigation into the impact of macrophages on myocardial fibrosis within the context of cardiac diseases. In this review, we systematically summarize the diverse phenotypic and functional plasticity of macrophages in regulatory mechanisms of cardiovascular diseases and unprecedented insights introduced by single-cell sequencing technologies, with a focus on different causes and characteristics of diseases, especially the relationship between inflammation and fibrosis in cardiac diseases (myocardial infarction, pressure overload, myocarditis, dilated cardiomyopathy, diabetic cardiomyopathy and cardiac aging) and the relationship between inflammation and vascular injury in vascular diseases (atherosclerosis and aneurysm). Finally, we also highlight the preclinical/clinical macrophage targeting strategies and translational implications.

## Introduction

The immune response is an important driver of cardiovascular disease (CVD) occurrence and development. Macrophages are key immune cells that exert significant impact on the entire process from inflammation to repair in CVD by expressing specific phenotypes.^1–3^ Generally, upon injury, macrophages are massively recruited to the damaged area by C-C chemokine receptor type 2 (CCR2) and become the dominant immune cells. Macrophages not only play a major role in the inflammatory response by phagocyting tissue debris and releasing a large number of pro-inflammatory cytokines and proteinases, but also secrete a variety of mediators to stimulate extracellular matrix (ECM) production, cell proliferation and angiogenesis.^3,4^ In addition, macrophages occupy a central position and participate in cross-talk with other cells mainly through the release of different mediators, such as affecting the chemotaxis and functions of other immune cells to regulate immune response, facilitating or suppressing the generation of vascular endothelial cells (ECs) and regulating fibrosis by directly facilitating the activation and proliferation of fibroblasts and promoting their differentiation into myofibroblasts.^5^ In spite of these common features, there are differences in the phenotype and function of macrophages in specific disease contexts. For example, in the late stage of ischemic injury, resident macrophages tend to proliferate and play a repair role, whereas, in other cardiac diseases, recruited macrophages play a major role, with or without resident macrophage loss. In particular, macrophages in atherosclerosis (AS) phagocytose oxidized low-density lipoprotein (OxLDL) to form foam cells, which are mainly involved in lipid metabolism. Hence, it can be seen that macrophages are indispensable contributors to the development of various CVD.

Myocardial fibrosis, a common pathological outcome of various CVD, is characterized by excessive deposition and abnormal distribution of collagen. Macrophages play an important role in the occurrence, progression and repair of myocardial fibrosis. The structural quality, fibrillary composition and metabolic properties of fibrosis differ under diverse etiologies, resulting in distinct pathophysiological characteristics and clinical manifestations.^6,7^ Based on histopathological characteristics, fibrosis can primarily be classified into replacement fibrosis and interstitial fibrosis.^4^ After myocardial ischemic injury, cardiomyocyte death and replacement fibrosis occur, leading to systolic dysfunction. In non-ischemic injury, interstitial fibrosis mainly occurs, contributing to diastolic dysfunction.^6^ Therefore, it is necessary to consider the diverse disease contexts and types of fibrosis separately when investigating fibrotic pathways. Inflammation is also the main feature of vascular diseases, which can give rise to thrombosis, hardening and narrowing of blood vessel walls and CVD such as myocardial infarction (MI).^8^ Thus, for effective CVD therapy, identifying and targeting cells along with molecules that regulate fibrosis and inflammation becomes imperative in order to limit or reverse their overdevelopment without disrupting tissue repair. Besides, with the development of emerging technologies such as single-cell RNA sequencing (scRNA-seq), the cellular heterogeneity, microenvironmental signaling, and intracellular regulation during the process of CVD have been elucidated to a greater extent.^9,10^ For the first time, we comprehensively summarize macrophage classifications and the mechanisms by which macrophages regulate the development of CVD in a range of contexts, including ischemic cardiac injury (acute myocardial infarction (AMI), ischemia-reperfusion injury (IRI), and chronic myocardial infarction (CMI)), non-ischemic cardiac injury (pressure overload (PO), myocarditis, dilated cardiomyopathy (DCM), diabetic cardiomyopathy, and cardiac aging) and vascular diseases (AS and aneurysms), where we concentrate on macrophage-regulated fibrosis formation in cardiac diseases. In addition, we propose the heterogeneity of macrophages from a single-cell perspective and provide new insights into the complex biological processes underlying macrophage-mediated CVD. Finally, aimed at providing new intervention targets and therapeutic strategies for the clinical treatment of CVD, preclinical strategies and published/ongoing clinical trials targeting macrophages are further consolidated.

## Origin, phenotype, and function of macrophages in cardiovascular system

The traditional view holds that macrophages are derived from circulating monocytes and are classified into M1/M2 macrophages based on the different stimuli required for in vitro culture. M2 macrophages can be subdivided into four subsets: M2a, M2b, M2c, and M2d.^11^ M1 macrophages highly express markers such as cluster of differentiation (CD) 80, CD86, and inducible nitric oxide synthase (iNOS), which are primarily associated with the inflammatory response.^12^ M2 macrophages highly express markers such as CD163, CD206, Arg1, FIZZ1, and YM1. In addition to the M2b subset, which secretes both pro-inflammatory and anti-inflammatory factors to regulate the immune response, other M2 subsets exhibit a repair phenotype mainly through the secretion of anti-inflammatory and pro-fibrotic factors.^11^ Since there are multiple influencing factors in vivo, the extreme classification of M1/M2 macrophages cannot summarize the complex and diverse functions of macrophages.^13,14^ In recent years, macrophages have been defined and classified as tissue-resident macrophages and monocyte-derived macrophages according to their different origins, and they have distinct phenotypes and functions. Referring to tissue-specific subsets that differentiate during organogenesis and are capable of establishing stable spatial and functional relationships with specific tissue cells, tissue-resident macrophages are mainly of embryonic origin and equipped with strong self-renewal, anti-inflammatory, and homeostasis maintenance abilities.^15^ Circulating monocyte-derived macrophages are primarily pro-inflammatory and actively produce high levels of pro-inflammatory cytokines and chemokines.

### Origin, phenotype and function of macrophages in heart

**Fig. 1:**
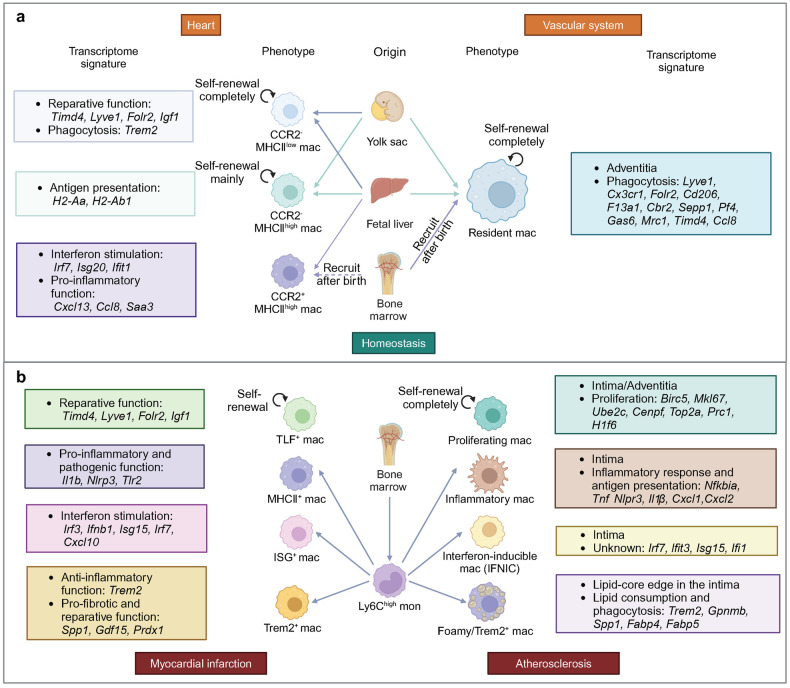
Origin, phenotype and function of macrophages in cardiovascular system under homeostasis, MI and AS. **a** In cardiac homeostasis, three types of resident macrophages exist in the heart. CCR2^-^MHC^low^ macrophages and CCR2^-^MHC^high^ macrophages are derived from yolk sac cells and fetal liver monocytes and maintain the number of subpopulations through self-renewal, while monocytes also contribute a small amount to the number of subpopulations. CCR2^+^MHC^high^ macrophages are derived from fetal liver monocytes and are gradually replaced by circulating monocytes during development. Artery-resident macrophages, predominantly located in the adventitia during homeostasis, are derived from yolk sac cells, fetal liver monocytes and bone marrow (after birth). Main functions and transcriptome signature of each subset are highlighted in the colored corresponding boxes. **b** When MI occurs, cardiac TLF^+^ macrophages undergo self-renewal. In addition, a large number of Ly6C^high^ monocytes infiltrate into the heart and mainly differentiate into three types of macrophages, including MHC^+^ macrophages, ISG^+^ macrophages and Trem2^+^ macrophages. In AS, macrophages can be classified into four main subsets, including proliferating macrophages, inflammatory macrophages, IFNIC and foamy/TREM2^+^ macrophages. Proliferating macrophages maintain the number of subpopulations through completely self-renewal and other subsets are derived from Ly6C^high^ monocytes. Main location, functions and transcriptome signature of each subset are highlighted in the colored corresponding boxes. (Created with BioRender.com)

In recent years, CCR2 expression, which can reflect the dynamic changes in macrophage phenotype and the origin of cardiac macrophages, has been utilized for the classification of cardiac macrophages.^16–18^ CCR2^-^ macrophages derived from embryonic yolk sac and fetal liver monocytes are maintained in the absence of monocyte recruitment, whereas CCR2^+^ macrophages are sustained through monocyte recruitment. Among them, CCR2^-^ subset and a few CCR2^+^ subsets are resident macrophages. In addition, resident and recruited macrophages in the heart also express major histocompatibility complex class II (MHC-II)/human leukocyte antigen-DR (HLA-DR) to varying degrees, which are associated with antigen presentation and the activation of T cells.^19^ The introduction of MHC-II markers allows for better differentiation of macrophage subsets with distinct functions. Based on CCR2 and MHC-II/HLA-DR markers, mouse cardiac macrophages can be categorized into three subsets: CCR2^-^MHCII^low^, CCR2^-^MHC-II^high^, and CCR2^+^MHC-II^high^ (Fig. 1a). Human cardiac macrophages can be categorized into two subsets: CCR2^-^HLA-DR^high^ and CCR2^+^HLA-DR^high^.^16,20^ The CCR2^+^MHC-II^high^ subset replaces CCR2^-^ macrophages during aging and myocardial injury.^16,18,20,21^ Notably, MHC-II markers in embryonic-derived macrophages are gradually upregulated after birth, which first appear in the CCR2^+^ subset and then in the CCR2^-^ subset.^18^ Thus, the majority of cardiac macrophages in neonatal mice are CCR2^-^MHC-II^low^ subset, whereas the adult mouse heart contains three resident macrophage subsets.^21^

Different subsets of macrophages focus on specific functions.^3^ The functions of resident macrophages include secreting anti-inflammatory mediators, promoting tissue repair, clearing apoptotic cells and damaged mitochondria, regulating myocardial fibrosis and inhibiting hypertrophy. In cardiac diseases, monocytes are recruited to lesion sites via C-C motif chemokine ligand (CCL) 2/ C-X3-C motif chemokine ligand 1 (CX3CL1) and predominantly differentiate into the CCR2^+^MHC-II^high^ macrophage subset.^22^ The effects of recruited macrophages on cardiac function and cardiac remodeling would be deeply discussed in the following context given diverse functions in relation to specific pathological states. In particular, MHC-II^high^ subset macrophages pivotally involve in immunodetection by scavenging the environment, recognizing and clearing pathogens, and presenting antigens.^3,22^ The distinct roles of specific macrophage subsets in myocardial fibrosis vary across different diseases, resulting in bidirectional regulatory effects on myocardial fibrosis^23^ (Table 1). When it comes to promoting fibrosis, firstly, macrophages secrete a variety of pro-fibrotic mediators, such as transforming growth factor-β (TGF-β), platelet-derived growth factor (PDGF), interleukin (IL)-10, vascular endothelial growth factor (VEGF), and amphiregulin (AREG), which directly induce the proliferation and activation of fibroblasts through the fibroblast receptors PDGFR, TGF-βR, and epidermal growth factor receptor (EGFR), thereby facilitating collagen synthesis.^24–26^ Furthermore, macrophages secrete substances that inhibit the degradation of the ECM, such as tissue inhibitor of matrix metalloproteinases (TIMPs), facilitating cardiac scar formation and myocardial remodeling.^27^ Additionally, macrophages have the potential to differentiate into fibroblasts and secrete collagen fibers, but the specific subset of macrophages with this capability remains unidentified.^28–30^ When referring to anti-fibrosis, some macrophages invovled can not only express a large number of matrix metalloproteinases (MMPs) but also stimulate other cells to produce MMPs, effectively degrading ECM components, which is crucial for the regression of fibrosis.^31^ Additionally, with a constant number of macrophages, it is generally believed that the polarization of macrophages towards the M2 phenotype can suppress inflammation over time, leading to a reduction in fibrosis.^32,33^ Macrophages can also regulate fibrosis through phagocytosis and modulation of inflammatory responses. While suppressing fibrosis by removing necrotic tissue and temporary matrix through phagocytosis,^31^ some pro-inflammatory or anti-inflammatory factors secreted by macrophages can directly act on interleukin 1 receptor (IL-1R), IL-6R complex, and angiotensin-II type 1 receptor (AT1R) on fibroblasts, or induce the increase of pro-fibrotic factors, thereby promoting fibrosis.^25,26,34–36^ In the regulation of both pro-repair fibroblasts and pro-fibrotic myofibroblasts, macrophages play a crucial role in maintaining a delicate balance, primarily through the secretion of inflammatory mediators. A majority of these pro-inflammatory mediators, including IL-1β, IL-6, and IL-23, which are released by macrophages, induce fibrosis and unfavorable cardiac remodeling in both ischemic and non-ischemic cardiac diseases.^7,37^ However, in the context of ischemic cardiac disease, which necessitates the production of replacement scar, early inflammation is advantageous for preserving cardiac repair.^38^ Conversely, macrophages aid in resolving chronic inflammation in cardiac disease through the process of phagocytosis and the secretion of anti-inflammatory mediators such as TGF-β and IL-10, thereby promoting cardiac repair.^39^ It is worth noting that different macrophage subsets may partially exhibit similar functions under M1/M2 classification and CCR2 classification. CCR2^+^ macrophages primarily display pro-inflammatory characteristics akin to those of M1 macrophages, but they can transition to a phenotype resembling the M2 subset during later stages of injury. The CCR2^-^ macrophages have comparable anti-inflammatory and reparative functions to M2 macrophages.

**Table 1:** Selected published articles related to the myocardial fibrosis regulated by macrophages

Study	Macrophage phenotype	Model	Main intervention	Effect of intervention on fibrosis	Effect of intervention on prognosis	Conclusion	Citation
**Acute myocardial infarction acute phase**							
Ducharme et al. (2000)	CD11b^+^	Permanent coronary artery ligation	Mmp9^−/−^	Inhibit	Improve	MMP-9 promotes macrophage recruitment into the heart and regulates the levels of other MMPs, which facilitates left ventricular dilation and collagen deposition after MI.	^78^
Hwang et al. (2001)	CD11b^+^	Permanent coronary artery ligation	IL-1β antibody treatment	Inhibit	Deteriorate	Administration of anti-IL-1β neutralizing antibodies during the acute phase of MI is harmful, leading to reduced collagen accumulation in the infarct area and promoting ventricular rupture and dilation. In the chronic phase of MI, anti-IL-1β antibodies inhibit collagen deposition in non-infarcted areas and reduce interstitial fibrosis.	^578^
Bujak et al. (2008)	CD11b^+^	Transient coronary artery ligation	Il1r1^−/−^	Inhibit	Improve	IL-1 signaling promotes the infiltration of neutrophils and macrophages into the infarcted myocardium and the expression of MMP-2, MMP-3, and TGF-β in the peri-infarct area through IL-1R1, thereby promoting inflammation and collagen deposition.	^65^
Howangyin et al. (2016)	F4/80^+^	Permanent coronary artery ligation	1. Mertk^−/−^/ Mfge8^−/−^ 2. *LysM*-Cre^+^/ Vegfa^fl/fl^	Both promote	Both deteriorate	Macrophages expressing Mertk and Mfge8 participate in the clearance of damaged tissue after MI. The endocytosis of damaged tissue promotes macrophage M2 polarization and secretes VEGFA to regulate neovascularization and collagen deposition.	^84^
Wang et al. (2017)	CD68^+^	Permanent coronary artery ligation	MiR-155^−/−^	Promote	Improve	Macrophage-derived miR-155 promotes fibroblast-mediated inflammation by reducing Socs1 expression and inhibits cardiac fibroblast proliferation by reducing Sos1 expression.	^588^
Bageghni et al. (2019)	CD11b^+^	Permanent coronary artery ligation	*Col1a2-*CreERT/ Il1r1^fl/–^	Inhibit	Improve	Specific knockdown of IL-1R1 on fibroblasts reduces the expression of cardiac remodeling markers and collagen deposition.	^70^
Jing et al. (2019)	CD11b^+^	Permanent coronary artery ligation	Il6^−/−^	Inhibit	Improve	IL-6 may promote collagen production by promoting the expression of TNF-α and inhibiting M2 macrophage activation.	^73^
Kubota et al. (2019)	Ly6C^low^	Permanent coronary artery ligation	Mmp12^−/−^	Inhibit	Deteriorate	MMP-12 produced by Ly6C^low^ macrophages prevents neutrophil infiltration by inhibiting the CXCL1/CXCL2/CXCL5-CXCR2 axis, thereby significantly reducing MMP-9 secretion and increasing collagen deposition.	^81^
Razin et al. (2021)	CD11b^+^	Permanent coronary artery ligation	Il1a^−/−^	Promote	Deteriorate	IL-1α stimulates fibroblasts to express StAR, thereby inhibiting fibroblast apoptosis during the inflammatory phase, which may be beneficial for inhibiting fibrosis.	^589^
Lugrin et al. (2023)	CD11b^+^	Permanent coronary artery ligation	Il1a^−/−^	Inhibit	Improve	IL-1α promotes the release of pro-inflammatory mediators such as IL-6 and MCP-1 and the expression of fibrotic genes such as CTGF, thereby promoting myocardial fibrosis.	^75^
**Acute myocardial infarction reparative phase**							
Bujak et al. (2007)	F4/80^+^	Permanent coronary artery ligation	Smad3^−/−^	Inhibit	Improve	Smad3 does not alter the time course of resolution of inflammation in healing infarcts but can promote interstitial fibrosis in non-infarcted myocardium to worsen cardiac function.	^103^
Krishnamurthy et al. (2007)	CD68^+^	Permanent coronary artery ligation	1. Recombinant IL-10 treatment 2. HuR knockdown by siRNA	Both inhibit	Both improve	IL-10 inhibits fibrosis by inhibiting the HuR/MMP-9 axis.	^112^
Dobaczewski et al. (2010)	F4/80^+^	Transient coronary artery ligation	Smad3^−/−^	Inhibit	NA	Knocking out Smad3 inhibits fibrosis mediated by TGF-β1 and CTGF, which results in an increase in the number but functional defects of fibroblasts, thereby reducing collagen deposition.	^101^
Shirakawa et al. (2018)	Galectin-3^high^ CD206^+^	Permanent coronary artery ligation	Spp1^−/−^	Inhibit	Deteriorate	After MI, the IL-10-STAT3-Galectin-3 axis is important for macrophage M2 polarization and production of the profibrotic substance OPN, and OPN is almost produced by Galectin-3^high^ CD206^+^ macrophages.	^110^
Chen et al. (2019)	LyzM^+^	Permanent coronary artery ligation	*LyzM*-Cre^+^/ Smad3^fl/fl^	No significant change	Deteriorate	After MI, Smad3 in macrophages can mediate the acquisition of phagocytic phenotype and promote an anti-inflammatory transition, but it has no significant effect on myofibroblast density or collagen content.	^108^
Shirakawa et al. (2020)	Galectin-3^high^ CD206^+^	Permanent coronary artery ligation	M-CSF activator treatment	Promote	Improve	IL-10 and M-CSF synergistically activate STAT3 and ERK in cardiac macrophages to upregulate the expression of Galectin-3 and MerTK, leading to the functional maturation of cardiac macrophages and the production of profibrotic substance OPN.	^109^
Alonso-Herranz et al. (2020)	Lyz2^+^	Transient and permanent coronary artery ligation	*Lyz2*-Cre^+^/ Mmp14^fl/fl^	Inhibit	Improve	Macrophages promote endothelial-mesenchymal transition through MMP-14/TGF-β1/Smad2 after MI, leading to myocardial fibrosis.	^93^
Chen et al. (2022)	LyzM^+^	Permanent coronary artery ligation	*LyzM*-Cre^+^/ Smad2^fl/fl^	No significant change	No significant change	Smad2 in bone marrow cells has no significant effect on the clearance of infarcted cells, inflammation or fibrosis in the infarcted heart.	^105^
Humeres et al. (2022)	F4/80^+^	Permanent coronary artery ligation	*Postn*-Cre^+^/ Smad7^fl/fl^	Promote	Deteriorate	The TGF-β-driven myofibroblast activation is regulated by negative feedback from Smad7 through inhibition of Smad2/3, ERK, AKT, and EGFR signaling.	^590^
Garlapati et al. (2023)	CCR2^+^	Permanent coronary artery ligation	1. *LysM*-Cre^+^/ F2rl1^fl/fl^ 2. *LysM*-Cre^+^/ F3^fl/fl^	Both inhibit	Both improve	TF-PAR2 signaling activates NOX2/ERK-dependent TGF-β1 production in myeloid cells and activates the TGF-β1/SMAD2 pathway to promote fibrosis.	^95^
Wang et al. (2023)	CD206^+^	Permanent coronary artery ligation	Vsig4^−/−^	Inhibit	Deteriorate	Hypoxia induces the expression of VSIG4 in macrophages, which promotes the expression of TGF-β1 and IL-10, leading to the transformation of fibroblasts into myofibroblasts.	^94^
**Chronic myocardial infarction**							
Yan et al. (2012)	CD11b^+^	Permanent coronary artery ligation	1. Il17a^−/−^ 2. Il23a^−/−^ 3. Tcrγδ^−/−^ 4. Tlr2^−/−^ 5. Tlr4^−/−^ 6. Tlr2^−/−^/ Tlr4^−/−^	All inhibit	All improve	TLR signaling regulates M1 macrophages to produce IL-1β and IL-23, which drive cardiac γδ T cell expansion and production of IL-17A. In the late stage of myocardial injury, IL-17A continues to promote the production of pro-inflammatory cytokines, MMPs, and TGF-β, stimulating fibroblast proliferation and collagen deposition.	^130^
Ismahil et al. (2014)	CCR2^+^	Permanent coronary artery ligation	1. Splenectomy 2. Splenocytes adoptively transferred	1. NA 2. Promote	1. Promote 2. Deteriorate	Spleen-derived proinflammatory macrophages and monocytes are increased in failing hearts, and they induce cardiac inflammation and fibrosis.	^118^
**Ischemia reperfusion**							
Huebener et al. (2008)	CCR2^+^	Transient coronary artery ligation	Cd44^−/−^	Inhibit	Deteriorate	After IR, CD44 expression is markedly induced in macrophages, and it increases collagen deposition by inhibiting post-infarction inflammatory response, stimulating the TGF-β signaling pathway, and promoting fibroblast infiltration and proliferation.	^139^
Fan et al. (2009)	F4/80^+^	Transient coronary artery ligation	Clec7a^−/−^	Inhibit	Improve	Dectin-1 induces macrophage M1 polarization and releases pro-inflammatory cytokines TNF-α, IL-1β, and IL-23. Dectin-1 also promotes the expression of CXCL1 and G-CSF in macrophages to mediate neutrophil infiltration, enhance early inflammatory response, and ultimately lead to more severe fibrosis.	^137^
Feng et al. (2022)	CCR2^+^	Transient coronary artery ligation	Ccl17^−/−^	Inhibit	Improve	CCL17 is expressed in CCR2^+^ macrophages and inhibits Tregs recruitment which can suppress macrophage-associated inflammation, promoting inflammation and fibrosis.	^138^
Li et al. (2023)	ARG-1^+^	Transient coronary artery ligation	M2-derived sEV treatment	Inhibit	Improve	sEV derived from M2 macrophages can regulate the glucose uptake and glycolysis levels of CCR2^+^ macrophages to reduce the production of mitochondrial reactive oxygen species, inducing the transformation of macrophages into a repair phenotype and ultimately promoting left ventricular fibrosis.	^142^
**Pressure overload**							
Ma et al. (2012)	F4/80^+^	Ang-II infusion	Il6^−/−^	Inhibit	NA	Macrophages stimulate cardiac fibroblasts to produce IL-6, which induces TGF-β1 production and Smad3 phosphorylation in cardiac fibroblasts, thereby stimulating myocardial fibrosis.	^184^
Verma et al. (2012)	F4/80^+^	ISO infusion	1. Il10^−/−^ 2. Recombinant IL-10 treatment	1. Promote 2. Inhibit	1. Deteriorate 2. Improve	IL-10 inhibits the NF-κB pathway through STAT3, thereby reducing isoproterenol-induced myocardial fibrosis.	^591^
Shimojo et al. (2015)	CCR2^+^	Ang-II infusion	Tnc^−/−^	Inhibit	NA	Tenascin-C accelerates the migration of macrophages and the expression of pro-inflammatory cytokines through the integrin αVβ3/NF-κB/interleukin-6 axis, thereby promoting the collagen secretion of cardiac fibroblasts.	^592^
Verma et al. (2017)	F4/80^+^	TAC	Il10^−/−^	Promote	Improve	IL-10 inhibits TGF-β-Smad-miR-21-mediated activation of bone marrow fibroblasts, thereby inhibiting fibrosis.	^174^
Khalil et al. (2017)	F4/80^+^	TAC	1. *Postn-*Cre^+^/ Tgfbr1/2^fl/fl^ 2. *Postn-*Cre^+^/ Smad2^fl/fl^ 3. *Postn-*Cre^+^/ Smad3^fl/fl^ 4. *Postn-*Cre^+^/ Smad2/3^fl/fl^	1. Inhibit 2. No significant change 3. Inhibit 4. Inhibit	All improve	TGF-β-Smad2/3 signaling in cardiac fibroblasts promotes fibroblast differentiation and proliferation to facilitate the fibrotic response induced by pressure overload.	^593^
Suetomi et al. (2018)	F4/80^+^	TAC	1. *MLC2v-*Cre^+^/ Camk2d^fl/fl^ 2. *α-MHC-*Cre^+^/ Ccl2^fl/fl^	Both inhibit	Both improve	CaMKIIδ activates the NF-κB pathway in cardiomyocytes, which activates the inflammasome and expresses inflammatory genes such as MCP-1 and IL-1β, leading to the recruitment of macrophages and ultimately fibrosis.	^594^
Chou et al. (2018)	CD11b^+^	Aldosterone infusion	1. MR inhibitor treatment 2. PI3K / Akt inhibitor treatment 3. MAPK / ERK inhibitor treatment 4. MAPK / p38 inhibitor treatment 5. IL-6 inhibitor treatment	All inhibit	NA	Aldosterone induces endothelial cells to produce IL-6 through the MR/PI3K/Akt/NF-κB pathway, and IL-6 promotes collagen secretion by fibroblasts through IL-6 trans-signaling.	^185^
Hulsmans et al. (2018)	CX3CR1^+^	Aldosterone infusion	*Cx3cr1-*Cre^+^/ Il10^fl/fl^	Inhibit	Improve	IL-10 produced by cardiac macrophages promotes the conversion of macrophages into MHC-II^high^ macrophages and the expression of more OPN and TGF-β and fewer MMPs, thereby promoting collagen deposition.	^170^
Abe et al. (2019)	Ly6C^high^	TAC	1. *LysM-*Cre^+^/ Hif1a^fl/fl^ 2. *Col1a1-*Cre^+^/Osmr^fl/fl^	Both promote	Both deteriorate	Ly6C^high^ macrophages accumulate in myocardial hypoxic areas in a HIF-1α-dependent manner and secrete oncostatin-m to directly inhibit TGF-β-mediated fibroblast activation.	^190^
Liao et al. (2020)	CD11b^+^	Aldosterone infusion	IL-6 antibody treatment	Inhibit	NA	Aldosterone promotes macrophage infiltration through the MR/IL-6/JAK/COX-2/PGE2 pathway, thereby promoting fibrosis.	^159^
Lv et al. (2021)	F4/80^+^	TAC	NLRP3 inhibitor treatment	Inhibit	Improve	NLRP3 promotes myocardial fibrosis by promoting macrophage infiltration and activating the TGF-β/Smad4 pathway.	^153^
Chen et al. (2022)	Ly6C^high^	Ang-II infusion	*LysM-*Cre^+^/ Wwp2^fl/fl^	Inhibit	Improve	The interaction of WWP2 with transcription factor IRF7 in macrophages can drive downstream CCL5 and IFN signaling to promote the infiltration of Ly6C^high^ monocytes and the expression of pro-inflammatory genes, thereby promoting myofibroblast activation.	^167^
Yu et al. (2023)	LysM^+^	TAC	*LysM-*Cre^+^/ Nlrc5^fl/fl^	Promote	Deteriorate	NLRC5 interacts with HSPA8 in cardiac macrophages to inhibit the NF-κB pathway and IL-6 secretion, thereby inhibiting cardiac fibroblast activation.	^169^
Ye et al. (2023)	F4/80^+^	Ang-II infusion	Clec7a^−/−^	Inhibit	Improve	Ang-II acts on Dectin-1 to activate the Syk/NF-κB signaling pathway and induce the expression of pro-inflammatory cytokines in macrophages, thereby activating fibroblasts.	^157^
**Myocarditis**							
Szalay et al. (2009)	Mac-3^+^	CVB3 infection	Vitamin D analog treatment	Inhibit	Improve	Calcitriol produced by vitamin D metabolism activates vitamin D signaling in macrophages, increases the expression of pERK in macrophages, and stimulates the production of pro-fibrotic substances such as OPN and TGF-β1.	^210^
Gruhle et al. (2012)	CCR2^+^	CVB3 infection	Iloprost treatment	Promote	Deteriorate	Infiltrating macrophages express iNOS to stimulate p44/42 MAPK activation, which promotes macrophages to secrete CTGF, ultimately leading to increased fibrosis.	^209^
Kraft et al. (2019)	Mac-3^+^	CVB3 infection	IL-1β antibody treatment	Inhibit	NA	The virus induces macrophages to secrete IL-1, which may stimulate an elevation in circulating levels of IL-6, thereby facilitating myocardial fibrosis.	^208^
**Dilated cardiomyopathy**							
Psarras et al. (2012)	CD11^+^	Desmin knockout	Spp1^−/−^	Inhibit	Improve	Infiltrating macrophages are the main source of OPN, and OPN can promote the secretion of Galectin-3 to promote fibrosis.	^220^
Touvron et al. (2012)	CCR2-	Cardiac-specific SRF knockout	Cardiomyocyte-specific IGF-1 overexpression	Inhibit	Improve	IGF-1 prevents fibroblast proliferation and myocardial fibrosis by inhibiting CTGF.	^223^
Zhang et al. (2021)	F4/80^+^	DOX infusion	NLRP3 inhibitor treatment	Inhibit	Improve	NLRP3 inflammasome promotes the activation of ASC, caspase-1, IL-18, IL-1β, and GSDMD, thereby promoting inflammation and myocardial fibrosis.	^218^
Liu et al. (2022)	CCR2^+^ CD206^+^	DOX infusion	M2-like macrophages infusion	Inhibit	Improve	Adoptive transfer of M2-like macrophages attenuates doxorubicin-induced myocardial fibrosis by transferring mitochondria from macrophages into injured cardiomyocytes.	^32^
**Diabetic cardiomyopathy**							
Qi et al. (2014)	F4/80^+^	Ang-II infusion	Adipoq^−/−^	Promote	Deteriorate	APN level is significantly reduced in diabetes, which reduces macrophage autophagy and increases the secretion of inflammatory cytokines, thereby promoting myocardial fibrosis.	^243^
Govindappa et al. (2020)	CCR2^+^	Obese receptor knockout	Bone marrow-derived macrophages-exosomes with HuR deficiency	Inhibit	Improve	Exosome-associated HuR from bone marrow-derived macrophages is transferred to fibroblasts and induces inflammatory and fibrotic responses in fibroblasts.	^240^
Widiapradja et al. (2021)	Galectin-3^+^ CD86^+^	Obese receptor knockout	SP treatment	Inhibit	Improve	Reduced SP in diabetic hearts significantly increases M1/M2 ratio, leading to the occurrence of fibrosis.	^239^
Wu et al. (2022)	Galectin-3^+^ CD86^+^	Streptozotocin-induced and intermittent high-glucose infusion	SGLT1 knockdown by shRNA	Inhibit	Improve	Glycemic variability promotes macrophages polarization toward M1 by acting on SGLT-1, thereby aggravating myocardial fibrosis.	^238^
Zhu et al. (2022)	CD68^+^	Streptozotocin-induced	Galectin-3 knockdown by shRNA	Inhibit	Improve	High glucose induces an increase in Galectin-3 in macrophages. Galectin-3 secretes pro-inflammatory cytokines by activating NF-κB to promote myocardial fibrosis.	^237^
Yang et al. (2023)	F4/80^+^	Streptozotocin-induced	Clec7a^−/−^	Inhibit	Improve	High glucose increases the expression of macrophage pattern recognition receptor Dectin-1. Dectin-1 secretes pro-inflammatory cytokines by activating NF-κB and promotes myocardial fibrosis.	^236^
**Cardiac aging**							
Trial et al. (2017)	CD36^+^	Natural aging	Ccl2^−/−^	Inhibit	Improve	Fibroblasts in the aging heart highly express MCP-1 in response to ROS. MCP-1 induces monocyte infiltration and polarization into alternatively activated M2a macrophages, thereby promoting fibrosis.	^250^
Toba et al. (2017)	F4/80^+^	Natural aging	Macrophage-specific Mmp9 overexpression	Inhibit	Deteriorate	With age, overexpression of macrophage-derived MMP-9 leads to insufficient angiogenesis and then triggers myocardial inflammatory response, which induces the production of fibrotic cytokines and promotes the accumulation of collagen.	^255^
Cieslik et al. (2017)	CD36^+^	Natural aging	AMPK activator treatment	Inhibit	Improve	The Erk pathway is activated in fibroblasts in the aging heart to promote MCP-1 secretion. MCP-1 then mediates monocyte infiltration and polarization into M2a macrophages, promoting myocardial fibrosis.	^251^

*TGF* transforming growth factor, *Mertk* Mer tyrosine kinase, *Mfge8* Milk fat globule epidermal growth factor 8, *VEGFA* vascular endothelial growth factor A, *Socs1* suppressor of cytokine signaling 1, *Sos1* son of sevenless homolog 1, *Ly6C* lymphocyte antigen 6 complex, locus C, *StAR* steroidogenic acute regulatory protein, *CTGF* connective tissue growth factor, *Smad* small mothers against decapentaplegic, *HuR* human antigen R, *NA* not applicable, *OPN* osteopontin, *STAT3* signal transducers and activators of transduction 3, *LyzM* lysozyme M, *M-CSF* macrophage colony-stimulating factor, *ERK* extracellular signal-regulated kinase, *Lyz2* lysozyme M, *EGFR* epidermal growth factor receptor, *TF* tissue factor, *PAR2* protease-activated receptor 2, *NOX2* NADPH oxidase 2, *VSIG4* V-set and Ig domain-containing 4, *Dectin-1* dendritic cell-associated C-type lectin-1, *G-CSF* granulocyte colony-stimulating factor, *sEV* small extracellular vesicles, *TLR* toll-like receptor, *ISO* isoproterenol, *TAC* transverse aortic constriction, *CaMKIIδ* Calcium/calmodulin dependent protein kinase IIδ, *MR* Mineralocorticoid receptor, *PI3K* Phosphatidylinositol-3-kinase, *MAPK* Mitogen-activated protein kinase, *HIF-1α* hypoxia-inducible Factor-1α, *JAK* Janus kinase, *COX-2* cyclooxygenases-2, *NLRP3* NOD-like receptor thermal protein domain associated protein 3, *WWP2* WW domain-containing protein 2, *IRF7* interferon regulatory factor 7, *IFN* interferon, *NLRC5* NLR family CARD domain containing 5, *HSPA8* heat shock protein family A member 8, *Syk* Spleen tyrosine kinase, *CVB3* Coxsackievirus B3, *iNOS* Inducible nitric oxide synthase, *SRF* Serum response factor, *IGF-1* Insulin-like growth factor 1, *DOX* doxorubicin, *ASC* apoptosis-associated speck-like protein containing a caspase recruitment domain, *GSDMD* gasdermin D, *APN* adiponectin, *SP* substance P, *SGLT-1* sodium–glucose cotransporter 1, *AMPK* adenosine monophosphate-activated kinase

### Origin, phenotype and function of macrophages in vascular system

Artery-resident macrophages are predominantly distributed in the adventitia during homeostasis and have been found to originate from two main sources in mice. In the embryo, macrophages mainly develop from yolk sac-derived C-X3-C motif chemokine receptor 1 (CX3CR1)^+^ endothelial microparticles (EMPs), with a smaller contribution from fetal liver monocytes.^40^ After birth, these macrophages are immediately colonized and replaced by monocyte-differentiated macrophages. In adulthood, only about 20% of arterial-resident macrophages are still yolk sac-derived.^40^ Artery-resident macrophages express the CD206 marker.^41–43^ However, unlike cardiac macrophages, the CCR2^+^ subset also exists in artery-resident macrophages. In common with cardiac macrophages, arterial macrophages in neonatal mice are MHC-II^low^ and develop MHC-II^high^ macrophages after a period of birth.^40^ Independent of the replenishment of circulating monocytes, adult mouse artery-resident macrophages are sustained primarily through self-renewal. In addition, embryonic and monocyte-derived arterial macrophages have comparable self-renewal abilities^3,40^ (Fig. 1a). Although mouse vascular macrophages have been extensively studied, there is still limited data available on the origin and phenotype of human vascular macrophages. Nowadays, numerous studies are dedicated to mapping the development and differentiation of human vascular macrophages using high-precision single-cell transcriptome sequencing technology. It is found that the categorization of arterial macrophages at the single-cell level is cross-correlated with the traditional M1/M2 categorization.

Generally speaking, macrophages play an essential role in regulating phagocytosis, immune surveillance, inflammation and remodeling in blood vessel^3^ (Table 2). The phagocytosis and immune surveillance functions of macrophages are primarily performed by resident macrophages. Inflammatory response and vascular remodeling occur in vascular diseases. Macrophage proliferation in the early stages of vascular disease mainly depends on monocyte recruitment and differentiation.^40,44,45^ Among them, lymphocyte antigen 6 complex, locus C (Ly6C)^high^ monocytes chiefly differentiate into M1 macrophages, while it remains unclear which macrophage subpopulation Ly6C^low^ monocytes preferentially differentiate into. In terms of inflammation, macrophages facilitate chronic vascular inflammation by releasing pro-inflammatory cytokines such as IL-1, IL-6, and tumor necrosis factor (TNF). Mainly, inflammatory macrophages act similarly to the M1 phenotype.^8,46^ With respect to anti-inflammation, macrophages secrete anti-inflammatory factors, such as IL-10 and TGF-β, to suppress inflammation, similar to the M2 phenotype.^46–49^ A distinct population of foam cells in AS serves as an early marker of atherosclerotic plaques in mice and humans, which exhibit lipid phagocytosis and metabolic functions.^50,51^ However, dead foam cells release lipids and tissue factors to form the necrotic core, a crucial component of unstable plaques, which facilitates plaque rupture and subsequent intravascular clot formation, ultimately leading to MI.^52^ Macrophages also exhibit a high degree of matrix-degrading activity by releasing MMPs, which leads to the degradation of collagen.^53^ This process damages the vessel wall and results in adverse remodeling of the vessel wall.^53^ Furthermore, in addition to M1/M2 macrophages, recent studies have identified several novel macrophage subtypes in atherosclerotic plaques: Mox, M4, Mhem, and M(Hb) macrophages, which exhibit unique gene expression profiles and functional properties.^54^ Mox macrophages are bone marrow-derived cells with decreased expression of M1-M2 related genes, which can facilitate heme detoxification, reduce oxidative stress, and inhibit foam cell formation.^55^ M4 macrophages, mostly found in unstable plaques, highly express chemokines, such as CCL2 and CXCL4, and proteases, such as MMP-12, which recruit monocytes and neutrophils to degrade ECM proteins.^56^ In ruptured hemorrhage sites, M(Hb) and hemin-induced Mhem macrophages exist. Mhem, with a high expression of CD163 and heme oxygenase-1 (HO-1),^57–59^ promotes erythrocyte turnover by phagocytosis of senescent and damaged erythrocytes, thereby recycling iron and heme. M (Hb) highly expresses CD206 and CD163, which can remove free hemoglobin and inhibit its pro-oxidation effects.^60^

**Table 2:** Selected published articles related to vascular diseases regulated by macrophages

Study	Macrophage phenotype	Model	Main intervention	Effect of intervention on prognosis	Conclusion	Citation
**Atherosclerosis (Plaque progression)**						
Meurs et al. (2012)	CD68^+^	Ldlr^−/−^	Abcg1^−/−^	Progress (early stage) Stabilize (late stage)	The effect of ABCG1 on the development of AS lesions seems to depend on different stages, where ABCG1 has a protective effect in early lesions, while in late atherosclerosis attenuated apoptosis and compensatory mechanisms stimulate the development of late lesions.	^322^
Bhat et al. (2015)	CD68^+^	ApoE^−/−^	IL-18 treatment	Progress	IL-18 binds to IL-18 Rα via NF-κB to trigger an inflammatory cascade leading to plaque progression and destabilization. Blockade of NF-κB blocks IL-18 signaling by down-regulating IL-18, IL-18 Rα, CD36, and MMP-9, thereby reducing inflammation and restoring plaque stability by up-regulating LXR-α.	^292^
Tao et al. (2015)	CD68^+^	ApoE^−/−^	SR-B1^−/−^	Progress	SR-B1 deficiency in macrophages promotes defective efferocytosis signaling through the Src/PI3K/Rac1 pathway, leading to inflammation and increased plaque size.	^331^
Ceneri et al. (2017)	F4/80^+^	ApoE^−/−^	Rac2^−/−^	Progress	Macrophages rely on Rac1 to secrete IL-1β, and Rac2 prevents progressive calcification by inhibiting this pathway, thereby stabilizing plaques.	^351^
Oberoi et al. (2018)	CD68^+^	Ldlr^−/−^	TNF-α antibody	Progress	TNF-α antibody reduces circulating inflammatory markers while exhibiting no impact on body weight and glucose metabolism, but increases plasma triglyceride levels and pro-atherogenic VLDL cholesterol, as well as plaque burden in the thoracoabdominal aorta and aortic root.	^286^
Guo et al. (2018)	CD163^+^	ApoE^−/−^	CD163^−/−^	Stabilize	Through the CD163/HIF1α/VEGF-A pathway, CD163^+^ alternative macrophages promote plaque angiogenesis, vascular permeability and inflammation, which contributes to plaque progression.	^304^
Hettwer et al. (2021)	CD11b^+^	ApoE^−/−^	1. IL-1β antibody 2. NLRP3 inflammasome inhibition	Both stabilize	IL-1β and NLRP3 inflammasome induce leukocyte recruitment to atherosclerotic aortas, promote bone marrow hematopoietic stem cell proliferation and inflammatory response.	^276^
Singla et al. (2022)	LysM^+^	ApoE^−/−^	1. Sirpα^−/−^ 2. Cd47^−/−^ 3. Cd47^fl/fl^ LysM-Cre^+/-^	1. Stabilize 2. Stabilize 3. Progress	By inhibiting efferocytosis and the M2 macrophage phenotype, promoting cholesterol accumulation and oxidized LDL-induced inflammation, SIRPα or CD47 promotes plaque necrotic core formation. However, the opposite result is obtained with CD47-specific loss of myeloid cells.	^344^
**Atherosclerosis (Plaque rupture)**						
Souissi et al. (2008)	CD68^+^	NA	PPARα^−/−^	NA	By inhibiting MMP-12 expression in macrophages, PPARα agonists prevent inflammation and atherosclerotic plaque rupture.	^595^
**Atherosclerosis (Plaque regression)**						
van Gils et al. (2012)	CD68^+^	Ldlr^−/−^	Netrin1^−/−^	Regress	Through its receptor UNC5b, netrin-1 inhibits the migration of macrophages directed by chemokines CCL2 and CCL19, allowing macrophages to remain in the arterial wall to promote atherosclerosis.	^269^
Cardilo-Reis et al. (2012)	CD206^+^ and CD80^+^	Ldlr^−/−^	IL-13 treatment	Regress	IL-13 protects against atherosclerosis and contributes to a favorable plaque morphology by increasing collagen content, reducing VCAM-1-dependent monocyte recruitment and inducing M2 macrophage polarization.	^363^
Mueller et al. (2018)	CD11b^+^	ApoE^−/−^	LRP1^−/−^	Regress	Depletion of macrophage LRP1 enhances reverse cholesterol transport and increases the expression of the motility receptor CCR7 which drives macrophage egress from lesions, thus accelerating the regression of atherosclerosis.	^374^
Wang et al. (2018)	CD68^+^	Ldlr^−/−^	β-catenin^−/−^	Deteriorate	Inhibition of β-catenin triggers the downregulation of STAT3 and activation of STAT1 in macrophages, which leads to elevated macrophage inflammatory response and increased atherosclerosis.	^376^
**Aortic aneurysm (AAA)**						
Tazume et al. (2012)	CD68^+^	CaCl2-induced	Angptl2^−/−^	Improve	By inducing the expression of proinflammatory cytokines and MMP-9, macrophage-derived Angptl2 promotes aneurysm development and vascular destruction.	^405^
Hadi et al. (2018)	LysM^+^	Ang-II-induced	Ntn1^fl/fl^ LysM-Cre^+/-^	Improve	Acting via its receptor neogenin-1, netrin-1 induces the activation of VSMC and the expression of MMP-3, thereby promoting focal ECM degradation in AAA.	^466^
Yan et al. (2019)	MOMA-2^+^	Elastase-induced	1. IL-12p40 antibody 2. IL-23p19 antibody	Both improve	IL-12 and IL-23 released by macrophages promote macrophage expansion, MMP expression, Th1/Th17 cell differentiation and proliferation, thereby driving the chronic inflammatory response in AAA.	^596^
Wang et al. (2019)	CD68^+^	CaPO4-induced	exosome inhibitor	Improve	Macrophage-derived exosomes participate in the pathogenesis of AAA by inducing the expression of MMP-2 in VSMC through JNK and p38 pathways.	^422^
Yang et al. (2020)	Lyz2^+^	CaCl2-induced Ang-II-induced	Thbs1^fl/fl^ Lyz2-Cre	Improve	Inflammatory macrophages in AAA up-regulate the expression of TSP1, which enhances macrophage infiltration by inhibiting TIMP1 expression and leads to AAA progression.	^597^
Salarian et al. (2023)	CD11b^+^	Ang-II- induced	Mmp-12^fl/fl^ Csf1r-iCre	Deteriorate	By activating of complement activation and neutrophil extracellular trap pathway, absence of MMP-12 leads to more pronounced elastic layer degradation and reduced collagen integrity, and ultimately adverse aortic remodeling and death from rupture.	^598^
Davis et al. (2023)	Lys2^+^	Elastase-induced Ang-II-induced	Setdb2^fl/fl^ Lys2-Cre	Improve	Macrophages in AAA significantly up-regulate the expression of SETDB2, trimethylating histone 3 lysine 9 on the TIMP1-3 gene promoters, thereby suppressing TIMP1-3 transcription and leading to unregulated matrix metalloproteinase activity, ultimately contributing to vascular inflammation, macrophage infiltration and destruction of aortic structure.	^599^
Ye et al. (2024)	F4/80^+^	Ang-II-induced	1. Gsdmd^−/−^ 2. AAV-F4/80-shGSDMD	Both improve	Macrophages in AAA express GSDMD, which promotes AAA and aortic pyroptosis. GSDMD also promoted LPS^+^ nigericin-induced secretion of multiple cytokines.	^600^

*CD* cluster of differentiation, *Ldlr* low density lipoprotein receptor, *ABCG1* ATP-binding cassette transporter G1, *AS* atherosclerosis, *ApoE* apolipoprotein E, *IL* interleukin, *NF-κB* nuclear factor κB, *LXR* liver X receptor, *SR-B1* scavenger receptor class B type 1, *Src* proto-oncogene tyrosine-protein kinase, *PI3K* phosphatidylinositol-3-kinase, *Rac* Ras-related C3 botulinum toxin substrate, *TNF-α* tumor necrosis factor-α, *VLDL* very-low-density lipoprotein, *HIF1α* hypoxia inducible factor 1α, *VEGF-A* vascular endothelial growth factor A, *NLRP3* NOD-like receptor thermal protein domain associated protein 3, *SIRPα* signal regulatory protein α, *NA* not applicable, *PPAR* peroxisome proliferator-activated receptor, *MMP* matrix metalloproteinase, *CCL* C-C motif chemokine ligand, *VCAM-1* vascular cell adhesion molecule 1, *LRP1* lipoprotein receptor-related protein 1, *CCR7* C-C motif chemokine receptor 7, *STAT* signal transducers and activators of transduction, *Angptl2* angiopoietin-like protein 2, *Ang-II* angiotensin-II, *Ntn1* netrin-1, *VSMC* vascular smooth muscle cell, *ECM* extracellular matrix, *AAA* abdominal aortic aneurysms, *Thbs1* thrombospondin-1, *TSP1* thrombospondin-1, *TIMP1* tissue inhibitors of metalloproteinase, *Setdb2* SET domain bifurcated histone lysine methyltransferase 2, *GSDMD* gasdermin D, *LPS* lipopolysaccharides

## Heterogeneity and regulatory mechanisms of cardiac macrophages

### Ischemic injury

Ischemic injury directly leads to myocardial death, and since the regenerative capacity of cardiomyocytes is limited, repair of the infarcted heart mainly relies on scar tissue formation. There are three types of ischemic injury: acute ischemia, ischemia-reperfusion and chronic ischemia. In all three types of ischemia, the damaged cardiomyocytes and ECM release damage associated molecular patterns (DAMPs) to activate pattern recognition receptors (PRRs) on the surviving parenchymal cells, which secrete inflammatory cytokines and chemokines to recruit monocytes and other inflammatory cells.^38^ However, the types of cardiac remodeling and fibrosis induced by the three ischemic injuries differ. Acute ischemia mainly causes inflammation and replacement fibrosis at the infarct zone. Ischemia-reperfusion restores blood supply on the basis of acute ischemia, resulting in smaller infarct area and scar. The most important pathological process of chronic ischemia is interstitial fibrosis in the remote zone, which is induced by persistent chronic inflammation and altered cardiac structure.

#### Acute myocardial infarction

AMI is defined as the extensive death of cardiomyocytes and acute injury to the myocardium resulting from acute myocardial ischemia. Currently, the paradigm of MI in animal models is primarily divided into inflammatory, anti-inflammatory, and reparative stages.^24^ After MI, macrophages and other inflammatory cells are recruited to the infarct zone, leading to the production of pro-inflammatory cytokines to intensify inflammation and remove necrotic tissue.^38^ With the removal of necrotic tissue, macrophages switch phenotypes to produce anti-inflammatory cytokines that mediate the termination of inflammation and transition into the anti-inflammatory phase.^24^ Anti-inflammatory cytokines facilitate the differentiation of fibroblasts into myofibroblasts, which produce replacement fibrosis during the reparative phase.^38^ It should be noted that the high concentration of pro-inflammatory mediators prevents the pro-fibrotic mediators from exerting pro-fibrotic effects during the inflammatory phase,^61^ which may inhibit the premature emergence of collagen-producing cells, as the inflammatory phase is dominated by the clearance of infarct cells and matrix debris rather than collagen deposition.^61^ If inflammatory conduction is excessively blocked during the inflammatory phase, the risk of cardiac rupture leading to death and wall thinning leading to cardiac dilation increases, despite subsequent reductions in myofibroblast infiltration and collagen deposition.^62–64^ Unlike early intervention in inflammation to block the inflammatory cascade, late intervention in inflammation may primarily eliminate the direct effects of pro-inflammatory mediators on fibroblasts.^65^ In this review, we categorize the MI paradigm into two phases: the inflammatory phase and the reparative phase, discussing the fundamental principle that macrophages tend to secrete inflammatory cytokines during the inflammatory phase but anti-inflammatory cytokines to participate in scar formation during the reparative phase.

##### Inflammatory phase

The inflammatory phase is the period distinguished by recruitment of inflammatory cells and clearance of necrotic tissue, usually between 0 and 4 days after ischemia. Ly6C^high^ monocytes are recruited to the infarct zone through CCR2/CCL2 signaling and differentiate into CCR2^+^MHC-II^high^ macrophages, replacing the lost resident macrophages,^66,67^ so recruited CCR2^+^ macrophages play a dominant role in the inflammatory phase.^68^ When compared with tissue-resident macrophages, recruited CCR2^+^ macrophages express higher levels of inflammatory chemokines (monocyte chemoattractant protein-1 (MCP-1)), cytokines (IL-1β, IL-6, TNF-α), and genes implicated in adverse cardiac remodeling (MMP-9, TIMP-1).^20,21^ Different subsets of surviving resident macrophages play distinct roles in the process of recruiting monocytes. The tissue-resident CCR2^-^ macrophages can inhibit monocyte recruitment, playing an important role in preventing myocardial fibrosis after cardiac injury.^13,21^ The tissue-resident CCR2^+^ macrophages contribute to the recruitment of neutrophils and monocytes. Thereby, the depletion of this subset attenuates inflammation and myocardial fibrosis following MI.^13,69^

**Fig. 2:**
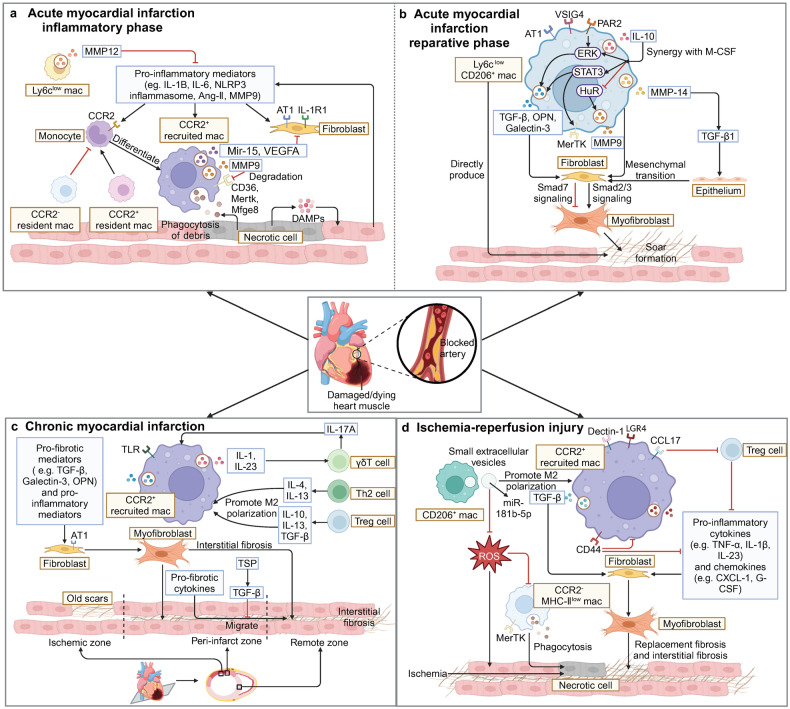
Regulations of myocardial fibrosis by macrophages after ischemic injury. **a** In the inflammatory phase of AMI, DAMP activates retained cells in the heart to release pro-inflammatory mediators, thereby promoting monocyte infiltration and differentiation into CCR2^+^ macrophages. CCR2^+^ macrophages secrete mediators (such as MMPs, miR-15, and VEGFA) to regulate inflammation and fibrosis in order to clear necrotic tissue and prepare for subsequent cardiac repair. **b** In the reparative phase of AMI, restorative Ly6C^low^CD206^+^ macrophages become the main macrophage subset in the heart. They secrete anti-inflammatory and pro-fibrotic mediators such as TGF-β, IL-10, galectin-3, and IL-10 to promote the conversion of fibroblasts into myofibroblasts which secrete collagen to form scars. **c** When CMI occurs, CCR2^+^ macrophages continue to infiltrate into the heart, interact with T cells, and secrete a large amount of pro-inflammatory and pro-fibrotic factors, causing interstitial fibrosis in remote zone. **d** When IRI occurs in the heart, a large number of CCR2^+^ macrophages accumulate in the early stage. They upregulate LGR4, Dectin-1 and CCL17 to promote inflammation and myocardial fibrosis, or upregulate the expression of CD44 and receive small extracellular vesicles secreted by M2 macrophages to convert to a reparative phenotype and attenuate the inflammatory response caused by ROS. (Created with BioRender.com)

Recruited macrophages clear necrotic tissue and create an environment conducive to scar repair through three mechanisms, including the synthesis of pro-inflammatory mediators, the synthesis of MMPs, and phagocytosis (Fig. 2a). These three mechanisms interact with each other, which is reflected in the fact that inflammation promotes the recruitment of macrophages to perform phagocytosis, phagocytosis promotes the normal progress of inflammation, and MMP is also involved in the regulation of substances related to inflammation and phagocytosis. Most of the pro-inflammatory mediators synthesized during the inflammatory phase play a pro-fibrotic role, including IL-1,^65,70^ NOD-like receptor thermal protein domain associated protein 3 (NLRP3) inflammasome,^16,71^ IL-6,^72,73^ and angiotensin-II (Ang-II),^74^ among which IL-1 plays a dominant role. IL-1 can be divided into IL-1α and IL-1β. IL-1α enhances the release of pro-inflammatory mediators such as IL-6 and MCP-1 and the expression of fibrosis genes such as connective tissue growth factor (CTGF), ultimately promoting myocardial fibrosis.^75^ Compared to IL-1α, IL-1β has contradictory effects. On the one hand, IL-1β secreted by recruited macrophages inhibits the expression of α-smooth muscle actin (α-SMA) in cardiac fibroblasts (CFs) and delays the transformation of myofibroblasts.^61^ On the other hand, IL-1β increases the fibrotic mediator TGF-β1 in the infarct zone and collaborates with TNF-α to increase the AT1R density on CFs, which prompts collagen deposition during the reparative phase.^36,65^ As an effector mediating pro-inflammatory signaling cascades in innate immunity, the caspase-recruitment domain family member 9 (CARD9) can upregulate the macrophages to express lipocalin 2 (Lcn2) and MMP-9, which consequently contributes to myocardial apoptosis, the deterioration of cardiac function and adverse remodeling after MI.^76^

MMP can affect fibrosis by regulating inflammatory signal transduction and degrading substrates dominated by ECM, but the former is the main function in the inflammatory phase.^77^ During the inflammatory phase, MMP-9, MMP-12, and MMP-28 are important MMPs secreted by macrophages, among which MMP-9 is more widely studied. MMP-9 exerts impacts on fibrosis mainly by affecting the expression of other MMP isoforms, including MMP-2, MMP-8, MMP-12, and MMP-13, to regulate the infiltration of macrophages and neutrophils.^78,79^ At present, it is not yet clear whether MMP-9 promotes or inhibits fibrosis, which may be due to the presence of multiple MMP subtypes involved. Unlike MMP-9, the fibrosis induced by MMP-12 and MMP-28 is beneficial for maintaining cardiac function in the later stage. Not only does MMP-12 stimulate the synthesis of cluster of differentiation (CD) 44 on the surface of neutrophils and enhance the interaction between CD44 and hyaluronan in the ECM, promoting the expression of apoptotic genes in neutrophils and the timely resolution of inflammation,^80^ but also downregulate the expression of C-X-C Motif Chemokine Ligand (CXCL) 1, CXCL2, and CXCL5 in the heart to prevent neutrophil infiltration and significantly reduce the secretion of MMP-9.^81^ MMP-28 can improve post-MI remodeling and dysfunction by inhibiting M2 macrophage activation, ECM synthesis, and collagen cross-linking.^82^

Phagocytosis consists of four processes: recognition, binding, internalization, and degradation of dying cells.^39^ After MI, apoptotic cells express “Find-me” and “Eat-me” signals (e.g., lipid mediators and nucleotides), which can attract macrophages and bind to phagocytosis-associated receptors on the cells, including myeloid-epithelial-reproductive receptor tyrosine kinase (Mertk), milk fat globule epidermal growth factor 8 (Mfge8), CD36, and legumain. Binding to these receptors initiates the phagocytosis process to remove neutrophils and myocardial debris,^83^ and inhibition of this process will lead to the obstruction of inflammatory program and greater fibrosis.^84^ The externalization of phosphatidylserine on the injured cell membrane is one of the earliest signals sufficient to activate the phagocytotic process, while Mertk- and Mfge8-expressing monocyte/macrophages play nonredundant roles in the recognition of phosphatidylserine,^84^ which mediates the clearance of damaged cardiomyocytes and favors the secretion of VEGFA to locally repair the dysfunctional heart.^84^ CD36, a scavenger receptor, is important for macrophage phagocytosis of apoptotic neutrophils. MI triggers macrophage infiltration into the infarct area to release abundant CXCL4, which decreases CD36 expression in a direct or MMP-9 dependent manner to inhibit macrophage phagocytosis of dead myocytes and neutrophils, eventually resulting in adverse remodeling post-MI.^83^ Resident macrophage-derived legumain promotes the efferocytosis of apoptotic cardiomyocytes, bringing about the recruitment of CCR2^+^ MHC-II^high^ macrophages and the inhibition of pro-inflammatory cytokine secretion, thereby improving cardiac repair.^85^ In addition, recent studies have revealed that mitochondrial metabolism has the potential to affect macrophage efferocytosis. Macrophage mitochondrial complex I deficiency can promote glycolysis and increase mitochondrial reactive oxygen species (ROS) production, which aggravates the early inflammatory response and impairs efferocytosis, thereby hindering the proliferation and activation of fibroblasts and scar formation after MI.^86^ Apart from collagen present in the interstitium, vascular rupture will lead to the deposition of coagulation-related fibrin, whose clearance is mainly related to CCR2^+^ macrophages.^87^

##### Reparative phase

The reparative phase refers to the process of gradual resolution of inflammation, proliferation of myofibroblasts, and scar formation after the inflammatory phase, usually between 4 and 14 days after the onset of MI. After 3 days of MI, anti-inflammatory mediators are gradually generated to suppress neutrophil infiltration, enhance macrophage phagocytosis of apoptotic neutrophils, and transform the CCR2^+^Ly6C^high^ recruited macrophages into reparative phenotypes.^84,88^ Except for the remaining macrophages in the inflammatory phase, Ly6C^low^ monocytes are recruited through CX3CR1/CX3CL1 signaling and differentiate into CCR2^-^Ly6C^low^ macrophages.^89,90^ It is generally accepted that reparative macrophages are characterized by low expression of Ly6C, CCR2, MHC-II, and high expression of CD206 and MerTK, as well as high expression of a series of anti-inflammatory and fibrosis-related genes, including Il10, hypoxia-inducible factor 1 α (Hif1a), Vegfa, insulin-like growth factor 1 (Igf1), secreted phosphoprotein 1 (Spp1), and Tgfb.^22,91,92^ Macrophages are capable of facilitating endothelial-to-mesenchymal transition,^93^ and certain macrophages undergo mesenchymal transition to adopt a fibroblast-like phenotype, directly contributing to collagen production.^28–30^ However, the specific macrophage subset equipped with the potential for fibroblast differentiation remains unidentified. Fibroblast-like macrophages express Acta2, type I collagen, fibroblast specific protein-1, prolyl-4-hydroxylase, and fibroblast activation protein and other markers, thereby secreting collagen and promoting fibrosis progression.^28–30^

Reparative macrophages regulate fibrosis mainly through fibrosis mediators and anti-inflammatory factors, of which TGF-β and IL-10 are garnering significant interest (Fig. 2b). Macrophages are an important source of TGF-β during the reparative phase, which can be induced by a variety of substances, such as hypoxia-induced V-set and Ig domain-containing 4 (VSIG4),^94^ tissue factor (TF)-protease-activated receptor 2 (PAR2) signaling,^95^ renin-angiotensin-aldosterone system (RAAS),^96^ MMP-14,^97^ and galectin-3.^98,99^ Expressed primarily in the peri-infarct zone,^7^ TGF-β predominantly transmits downstream signals through the small mothers against decapentaplegic (Smad) family,^100^ the most important of which targets fibroblasts through the TGF-β/Smad3 axis to motivate their migration, transdifferentiation and synthesis of collagen and fibronectin.^101–104^ Compared to Smad3, Smad2 plays a partial but limited role in conducting TGF-β signaling.^95,97,104,105^ The process of TGF-β-driven myofibroblast activation is also modulated by negative feedback from Smad7 through inhibition of Smad2/3, extracellular signal-regulated kinase (ERK), protein kinase B (Akt), and EGFR signaling.^106,107^ It is worth mentioning that Smad3 in macrophages contributes to the acquisition of an anti-inflammatory phenotype, yet it does not exert a marked impact on subsequent collagen deposition, demonstrating that fibrosis is not always in connection with inflammation.^108^ In view of anti-inflammatory factors, IL-10 is a pleiotropic cytokine and plays a differential role in the regulation of fibrosis. Hypoxia-induced VSIG4 promotes IL-10 expression in M2 macrophages, which ultimately accounts for the transformation of CFs into myofibroblasts.^94^ IL-10 also synergizes with macrophage colony-stimulating factor (M-CSF) to activate signal transducers and activators of transduction 3 (STAT3) and ERK in cardiac macrophages, which in turn elevates the expression of galectin-3 and MerTK, driving cardiac macrophage polarization and osteopontin (OPN) production.^109,110^ As a consequence, this process contributes to fibrosis.^111^ However, several studies have also found that IL-10 may play a role in inhibiting fibrosis. On the one hand, IL-10 can inhibit human antigen R (HuR)/MMP-9 signaling and activate the STAT3 to suppress collagen deposition.^112,113^ On the other hand, IL-10 stimulates myofibroblasts to enter a hyper-activated state represented by enriched hyaluronan levels and reduced collagen through the regulation of macrophage M2 polarization.^114^ In this state, myofibroblasts exhibit heightened proliferation, but collagen I secretion and collagen I–III ratio are reduced, thereby significantly attenuating myocardial fibrosis,^114^ which may imply that fibroblast activation does not necessarily represent increased collagen deposition.

Regardless of some progress, the heterogeneity of macrophages during the reparative phase has not been thoroughly explored,^13,67^ which impedes the further comprehension of the mechanisms by which reparative macrophages regulate fibrosis. In recent years, some studies on subsets have shed new light on the mechanism of fibrosis. During the reparative phase, the number of CCR2^-^ resident macrophages gradually increases, but the ratio of resident macrophages to recruited macrophages does not return to the pre-infarction level.^67^ Moreover, genes that confer critical repair functions on resident macrophages (T-cell immunoglobulin- and mucin-domain-containing molecule-4 (Timd4), lymphatic vessel endothelial receptor 1 (Lyve1), Igf1, etc.) are not adopted by recruited macrophages, suggesting that recruited macrophages cannot compensate for the depletion of resident macrophages.^67^ Even if monocytes can be transformed into peripheral-derived resident macrophages, the time window for effective protection of cardiac function may have been missed,^67^ which put emphasis on the significance of understanding the functions of resident macrophages and the heterogeneity of recruited macrophages for fibrosis and cardiac repair.

#### Chronic myocardial infarction

CMI refers to the persistent ischemic injury of the myocardium, which can be regarded as a subsequent stage of AMI and can deteriorate into heart failure (HF). The commonly used model for constructing CMI in mice is the ligation of the coronary artery for several weeks. Interstitial fibrosis in the remote zone stands out as a pivotal characteristic of CMI, resulting in elevated cardiac stiffness and impaired heart function.^115^ Prolonged ischemia and heightened mechanical stress on the non-infarcted myocardium induce infiltration of inflammatory cells and activation of pro-fibrotic cytokines in the remote zone.^115^ In addition, continuously activated cells in old scars secrete pro-fibrotic factors that might traverse the interstitial gaps to the remote zone, triggering the activation and proliferation of local fibroblasts and collagen deposition.^74^

Although cardiac macrophages in CMI shares the origin from recruited monocytes and local macrophage proliferation as in AMI to some extent, the prolonged ischemic and stressful environment adds fuel to the proliferation of macrophages. Exposed to chronic stress in CMI, the heart elevates the release of norepinephrine (NE) from sympathetic nerves.^116,117^ On the one hand, NE controls the release of hematopoietic stem and progenitor cell (HSPC) through β3-adrenergic signaling. On the other hand, it downregulates the expression of CXCL12 to decrease HSPC homing.^116,117^ The strong cardiosplenic axis has also been found in CMI, with an increase in the proliferation of HSPC and innate immune cells in the spleen.^117,118^ Expanded intramedullary and extramedullary hematopoiesis causes circulating monocytes to continuously proliferate and mobilize to the heart, resulting in macrophage infiltration. Besides recruited monocytes, the activation of the mitogen-activated protein kinase (MAPK) pathway induces local cardiac macrophage proliferation in response to increased ventricular wall tension caused by thinning left ventricular wall and ventricular dilatation.^117^

The pro-inflammatory phenotype macrophages exhibit in CMI is attributed to the reduced mitochondrial oxidative phosphorylation in myocardial tissues, which in turn promotes the anti-inflammatory functions of macrophages^38,119,120^ (Fig. 2c). Besides, the interactions between cardiac macrophages and increased T cells which are exerted via cytokines in CMI are of vital importance for regulating fibrosis. Increased release of the inflammatory factors IL-1β and TNF-α by macrophages accounts for the inflammation and fibrosis in the myocardium.^121,122^ IL-1β and TNF-α continuously stimulate the upregulation of AT1R on fibroblasts within the peri-infarct zone, thereby intensifying the pro-fibrotic effect.^36^ TNF-α induces distinct effects specific to the tumor necrosis factor receptor (TNFR), with TNFR1 exacerbating fibrosis in the remote zone while TNFR2 mitigating it, which may also offer insights into the negative results seen in clinical trials of TNF antagonists.^123^ In dealing with the effects of T cells on macrophage activation, T helper (Th) 2 cells and regulatory T cells (Tregs) are the main phenotypes involved in CMI.^124^ Th2 cells secrete IL-4 and IL-13, whereas Tregs secrete IL-10, IL-13 and TGF-β, all of which can stimulate macrophage M2 polarization, leading to the production of pro-fibrotic cytokines such as TGF-β, galectin-3, and MMP-9.^125–128^ Galectin-3, an emerging biomarker associated with fibrosis, has been found to correlate with the development and severity of HF. It promotes fibrosis by inducing fibroblast proliferation and differentiation into myofibroblasts, as well as inducing macrophage M2 polarization.^128,129^ In terms of the effects of macrophages on T cells activation, IL-1β and IL-23 primarily produced by M1 macrophages synergize with toll-like receptor (TLR) signaling to promote the expansion of γδ T cell and the production of IL-17A.^130^ Regardless of the fact that IL-17A is not involved in the early inflammatory response, it plays a role in the later stage of remodeling, by means of enhancing the infiltration of macrophages, the secretion of pro-inflammatory cytokines and MMPs, as well as fibroblast proliferation and pro-fibrotic gene expression, which facilitates fibrosis as a consequence.^130^ In addition, the selective endogenous expression of thrombospondin (TSP)-1, a TGF-β activator and angiogenesis inhibitor, may serve as a “barrier” in the peri-infarct zone. TSP-1 locally inhibits the synthesis of inflammatory cytokines and chemokines by activating TGF-β, which limits the infiltration of macrophages and myofibroblasts, as well as the extension of inflammatory response to the non-infarcted area.^131^

#### Ischemia–reperfusion injury

Owing to the exposure of the myocardium to oxidative stress, which exacerbates myocardial dysfunction and causes structural damage during the reperfusion phase, reperfusion following acute ischemia sometimes fails to restore myocardial function and instead results in IRI.^132^ IRI can also induce MI, but it typically causes a non-transmural infarction with fewer necrotic cells and a smaller infarct area, leading to a smaller scar. Ischemia and reperfusion collectively induce cardiac remodeling, encompassing replacement fibrosis and interstitial fibrosis.^133^ In IRI, the precise demarcation between inflammatory and reparative phases remains elusive, probably due to the rapid maturation of the fibrous scar. Studies tend to focus on CCR2^+^ macrophages infiltrating in the early stage of the injury, while paying less attention to reparative macrophages in the later stage.^133^ Although numerous findings suggest that there are shared mediators and pathways that regulate inflammation and fibrosis akin to the non-reperfused infarction,^7,38^ unique mechanisms also make a vast influence on reperfused infarction (Fig. 2d).

In the early stage of IRI, phagocytosis is primarily dominated by CCR2^-^MHC-II^low^ macrophages through MerTK.^91^ However, the hydrolysis of MerTK by ROS after IRI results in decreased levels of the anti-inflammatory mediators IL-10 and TGF-β, along with an increase in the pro-inflammatory mediators IL-1β and TNF-α, which eventually hinder the resolution of inflammation and cardiac repair.^91^ In accord with MerTK, AXL also mediates the phagocytosis of macrophages, but it is mainly expressed in MHC-II^high^ macrophages.^134^ AXL and TLR4 co-stimulate STAT1 signaling to direct a HIF-1α-dependent shift towards glycolytic metabolism in cardiac macrophages, thereby polarizing macrophages into inflammatory phenotypes and facilitating IL-1β secretion.^134^ While CCR2^+^ macrophages, recruited mainly through MCP-1, dominate the inflammatory and fibrotic responses in the early stage of IRI.^135^ Subsequently recruited macrophages can regulate inflammation and fibrosis through the expression of leucine-rich repeat-containing G protein-coupled receptor (LGR) 4,^136^ dendritic cell-associated C-type lectin-1 (Dectin-1),^137^ CCL17,^138^ and CD44.^139^ LGR4 orchestrates a pro-inflammatory phenotype in macrophages by enhancing activator protein-1 (AP-1) transcriptional activity via the protein kinase A (PKA) / cyclic AMP-responsive element binding protein (CREB) pathway mediated c-Fos, Fosl1, and Fosb transactivation, thereby aggravating the local myocardial inflammatory response.^136^ Dectin-1 is a PRR chiefly expressed on macrophages.^137^ On the one hand, Dectin-1 induces macrophage M1 polarization, giving rise to the release of pro-inflammatory cytokines, such as TNF-α, IL-1β, and IL-23. On the other hand, it upregulates CXCL1 and granulocyte colony-stimulating factor (G-CSF) in macrophages, which mediate neutrophil infiltration.^137^ Early augmented inflammatory responses contribute to the aggravation of myocardial injury and ultimately culminate in the development of more severe fibrosis. Notably, the long-term effect of G-CSF may aid in preventing fibrosis. In the early stage, G-CSF accelerates the uptake of necrotic tissue by expanding neutrophil and macrophage populations, and promotes the dissolution of collagen by upregulating the expression of myocardial MMPs.^140^ In the later stage, G-CSF decreases the population of macrophages to inhibit the ongoing inflammatory response.^140^ CCL17, a chemokine selectively expressed in CCR2^+^ macrophages, promotes inflammation and fibrosis by inhibiting Tregs chemotaxis, thereby relieving the suppressive effect of Tregs on pro-inflammatory macrophages.^138^ CD44 is a widely distributed glycoprotein that mediates various cell-to-cell and cell-matrix interactions. It inhibits post-infarction inflammatory responses through interactions with hyaluronic acid, stimulates the TGF-β signaling pathway, promotes fibroblast infiltration and proliferation, and ultimately enhances collagen deposition.^139^ In the late stage of IRI, CCL2 stimulates the transformation of CCR2^+^ macrophages into a reparative phenotype and releases TGF-β to promote fibrosis.^141^ When it comes to pro-repair CCR2^-^ macrophages, Li et al. unveiled their ability to produce small extracellular vesicles (sEVs). When taken up by CCR2^+^ macrophages, the sEVs microRNA (miR)-181b-5p regulates glucose uptake and glycolysis in macrophages while mitigating mitochondrial ROS generation, which promotes left ventricular remodeling and fibrosis by polarizing macrophages towards a reparative phenotype.^142^ As opposed to what are mentioned above, M2b macrophages are anti-fibrotic macrophages that inhibit fibroblast activation by regulating the MAPK signaling pathway.^143^

### Non-ischemic injury

The occurrence and development of fibrosis are similarly observed in non-ischemic injuries. Multiple stimuli can trigger fibrosis in the absence of ischemia through fibrotic signaling pathways in macrophages, including mechanical stress and RAAS activation in PO, ROS in DCM and cardiac aging, and metabolic impairments associated with hyperglycemia in diabetic cardiomyopathy, etc. In ischemic injury, inflammation usually precedes fibrosis in a sequential manner, while in non-ischemic injury, inflammation and fibrosis commonly coexist. In non-ischemic injury, interstitial fibrosis is a chronic and progressive epiphenomenon of the sustained repression of non-circumscribed, self-perpetuating inflammation and the concomitant chronic activation of pro-fibrotic stimuli.

#### Pressure overload

PO is a mechanical disorder that causes cardiac hypertrophy and myocardial fibrosis, with hypertension and valvular heart disease being its primary causes. Contrary to MI where cell death releases antigens, immune responses in PO may be initiated by DAMPs and endogenous cardiac neoantigens, and compensatory mechanisms such as myocardial fibrosis and hypertrophy are adopted in response to the increased load.^144,145^ PO can be divided into the compensation period and the decompensated period.^133^ CCR2^-^ macrophages play a dominant role in the compensation period, inhibiting fibrosis and myocardial hypertrophy.^146–148^ However, with the continuous infiltration of monocytes, monocyte-derived CCR2^+^ macrophages replace CCR2^-^ macrophages to play a dominant role in the decompensation period, promoting myocardial fibrosis and hypertrophy.^148–150^ Depleting CCR2^+^ macrophages as early as possible in the compensation period can mitigate myocardial fibrosis, while depletion of CCR2^+^ macrophages or splenectomy in the decompensation period fails to halt the development of fibrosis,^149^ which attaches significant importance to early regulation of CCR2^+^ macrophages.^148–150^ Nevertheless, most of the current research on PO focuses on the mechanism by which recruited macrophages regulate myocardial fibrosis and hypertrophy, while the mechanism of tissue-resident macrophages has not been thoroughly investigated.

**Fig. 3:**
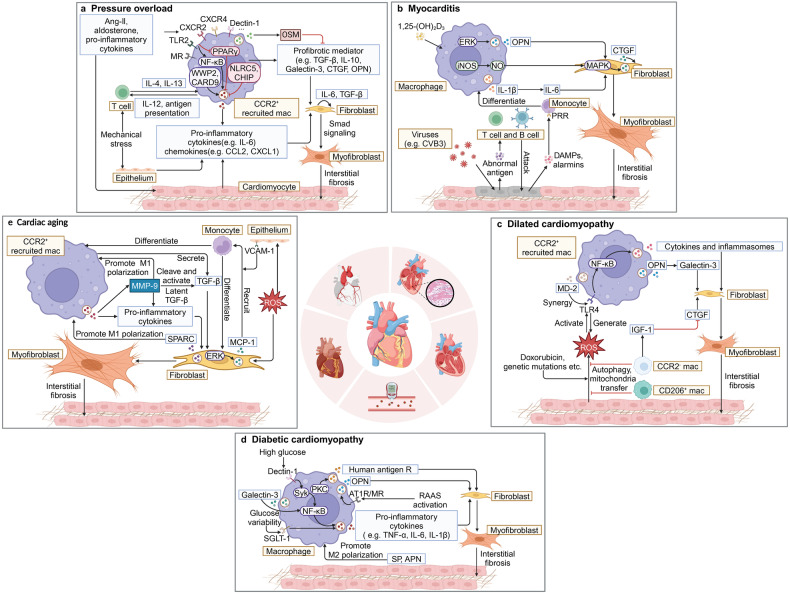
Regulations of myocardial fibrosis by macrophages after non-ischemic injury. **a** When PO occurs in the heart, Ang-II can recruit CCR2^+^ macrophages and cause them to secrete pro-inflammatory cytokines and chemokines such as IL-6, CCL2, and CXCL1. Mechanical stress directly or indirectly activates macrophages to exert pro-fibrotic functions. Interstitial fibrosis eventually develops in the heart. **b** When myocarditis occurs, viruses cause cardiomyocytes necrosis, and the necrotic cardiomyocytes produce DAMP which then recruits macrophages to the heart. Macrophages secrete mediators (such as OPN, NO, and IL-1β) that act on fibroblasts to promote the occurrence of myocardial interstitial fibrosis. **c** When DCM occurs, ROS activates CCR2^+^ macrophages to secrete pro-inflammatory and pro-fibrotic substances that act on fibroblasts, leading to interstitial fibrosis in the heart, while resident macrophages reduce myocardial fibrosis by improving cardiac metabolism or secreting the anti-fibrotic substance IGF-1. **d** When diabetic cardiomyopathy occurs, macrophages induce the production of pro-inflammatory substances through Dectin-1, Glectin-3, and SGLT-1, and release the fibrotic substances such as OPN and Human antigen R. Pro-inflammatory and pro-fibrotic substances jointly act on fibroblasts to promote the occurrence of interstitial fibrosis. **e** As cardiac aging occurs, increased ROS continues to promote monocytes to infiltrate into the heart and differentiate into CCR2^+^ macrophages. Macrophage-derived MMP-9 induces a series of pro-inflammatory and pro-fibrotic factors to act on fibroblasts, leading to interstitial fibrosis in the aging heart (Created with BioRender.com)

PO can be simulated by transverse aortic constriction (TAC) or Ang-II infusion. In both models, the onset of fibrosis and myocardial hypertrophy is mainly initiated by the neurohumoral system (mainly RAAS) and mechanical stress, while macrophage-mediated inflammation plays an important role in the subsequent progression of cardiac remodeling (Fig. 3a). Ang-II and aldosterone, which belong to RAAS, play a dominant role in PO-induced macrophage recruitment. Ang-II activates calcium/calmodulin-dependent protein kinase IIδ (CaMKIIδ) and initiates the nuclear factor-κB (NF-κB) pathway and inflammasome activation in cardiomyocytes, leading to CCR2^+^ macrophage recruitment.^151–153^ This process represents a potential initiating factor for PO. Ang-II also mediates macrophage recruitment through direct activation or up-regulation of chemokines to activate macrophage surface receptors TLR2,^154^ C-X-C motif chemokine receptor (CXCR) 2,^155^ CXCR4,^156^ Dectin-1,^157^ lymphocyte function-associated antigen 1 (LFA-1).^158^ Aldosterone promotes macrophage infiltration by activating mineralocorticoid receptor (MR), which may be mainly mediated by MR/IL-6/ cyclooxygenase-2 (COX 2) and MMP-1 and MMP-9 signaling pathways.^159^ Gamma-aminobutyric acid subtype A (GABA_A_) receptors, recognized as major neurotransmitter receptors in the central nervous system, have also been implicated to increase the number of Ly6C^low^ macrophages in the heart during PO and the number of circulating Ly6C^high^ monocytes during late PO, thereby favoring myocardial fibrosis and hypertrophy.^160^ In addition to the above common recruitment pathways, myocardial fibrosis and hypertrophy may each have some distinct recruitment pathways. Under sustained PO stimulation, sympathetic activation and subsequent intrarenal cell-to-cell interactions contribute to the expression and secretion of colony-stimulating factor 2 (CSF2). Nephrogenic CSF2 stimulates Ly6C^low^ macrophages in the heart to produce AREG and activate the cardiac hypertrophy program.^161^

Under the stimulation of the neurohumoral system, inhibition of peroxisome proliferator activated receptor γ (PPARγ) signaling and activation of NF-κB signaling in macrophages initiate downstream inflammasome activation and expression of inflammatory genes such as IL-1β, which eventually cause fibrosis^154–157^ and cardiac hypertrophy.^155,157,162,163^ Clonal hematopoiesis mediated by Tet2 mutations also accelerates cardiac hypertrophy and HF through the NLRP3/IL-1β pathway.^164,165^ In the context of Ang-II, CARD9 in macrophage cytoplasm also activates the NF-κB/MAPK signaling pathway and the expression of pro-inflammatory cytokines, thereby boosting fibrosis.^166^ Recently, Chen et al. have also revealed that WW domain-containing protein 2 (WWP2) in macrophages interacts with the transcription factor interferon regulatory factor (IRF)-7 to drive downstream CCL5 and interferon (IFN) signaling, which facilitates Ly6C^high^ monocyte infiltration and myofibroblast activation.^167^ However, some substances such as C terminus of Hsp70-interacting protein (CHIP), NOD-like receptor family caspase recruitment domain family domain containing 5 (NLRC5), and heat shock protein family A member 8 (HSPA8) in macrophages can inhibit macrophage recruitment and inflammatory gene expression to suppress the progression of fibrosis.^168,169^ Following the activation of MR on the surface of macrophages by aldosterone, macrophages secrete IL-10,^170^ galectin-3,^171^ CTGF,^172^ MMP-1, MMP-9,^159^ and other mediators that regulate fibrosis. It is worth noting that IL-10 showed opposite effects on fibrosis in different studies, which may be related to diverse sources of IL-10. Macrophage-derived IL-10 stimulates macrophage autocrine secretion of OPN and TGF-β, which activates fibroblasts.^170,173^ Conversely, systemically derived IL-10 may be beneficial for the inhibition of fibrosis, which is achieved by suppressing activation of bone marrow-derived fibroblasts mediated by the TGF-β-Smad-miRNA-21 pathway^174^ and blocking the NF-κB pathway,^175^ among other pathways.

Mechanical stress activates macrophages in both direct and indirect ways. Under the stimulation of mechanical stress, CCR2^+^ macrophages activate CD4^+^ T cells through antigen presentation,^144,150^ which produce TGF-β through integrin adhesion to CFs and induce myofibroblast transformation.^144,176^ The switch of CD4^+^ T cells to Th2 cells fosters M2 macrophage polarization, which promotes CF activation and myocardial fibrosis through TGF-β signaling.^177^ Mechanical stress activates fibroblasts to produce serum- and glucocorticoid-inducible kinase 1 (SGK1), chemokines, and other substances, which can induce macrophages to migrate and secrete pro-fibrotic mediators.^178^ Apart from indirect activation, mechanical stress can also directly induce M2 macrophage polarization by modifying macrophage morphology and actin cytoskeleton contractility,^179^ which may be associated with pro-fibrotic effects.^177^ Macrophages can also release secreted protein acidic and rich in cysteine (SPARC) to facilitate the processing of procollagen into insoluble fibrillar collagen, contributing to the development of fibrosis.^180^ In addition, macrophage migration inhibitory factor (MIF) antagonizes stress-induced cardiac hypertrophy and fibrosis responses by activating autophagy^181^ as well as maintaining a redox homeostasis phenotype.^182^ It is notable that the cardioprotective effect of CCR2^-^ macrophages is activated by mechanical stress in DCM.^183^

Inflammatory factors play a crucial role in adverse cardiac remodeling in PO. Among them, IL-6 related to fibrosis, IFN-γ and GATA3 related to myocardial hypertrophy have received more attention. Multiple studies based on PO models such as Ang-II infusion, aldosterone infusion, and TAC have found that IL-6 can recruit macrophages and directly activate fibroblasts, underscoring its pivotal role regulating fibrosis.^159,169,184,185^ Aldosterone and mechanical stress stimulate IL-6 synthesis by ECs, facilitating the recruitment of CCR2^+^ macrophages.^159,185–187^ In fibrosis regulation, CFs synthesize IL-6 in a macrophage-dependent manner,^184^ which induces the activation of TGF-β/Smad in CFs through IL-6 trans-signaling,^185^ thereby promoting the proliferation and differentiation of CFs.^153,154,184,188^ Among them, Smad3 signaling can also contribute to fibrosis by downregulating miR-25 and miR-29a.^189^ However, oncostatin M (OSM), a member of the IL-6 superfamily, plays a role in inhibiting fibrosis by directly preventing TGF-β-mediated CF from activation under hypoxic conditions.^190^ In terms of cardiac hypertrophy, IFN-γ is a common pro-inflammatory but anti-hypertrophic cytokine expressed in CD68^+^ macrophages, with the IFN-γ/Stat5 axis potentially mitigating PO-induced cardiac hypertrophy by activating the phosphatidylinositol 3-kinase (PI3K)/Akt pathway.^191^ Macrophage-derived GATA3 appears to facilitate PO-induced cardiac hypertrophy, possibly by regulating Th2 cell polarization and increasing the number of Ly6C^low^ macrophages.^192^

#### Myocarditis

Myocarditis is a pathological condition distinguished by the infiltration of inflammatory cells into the myocardium and the occurrence of non-ischemic necrosis in cardiomyocytes.^193^ Viruses are the primary inducing factors, among which coxsackievirus B3 (CVB3) is the most prevalent.^194^ Upon infection with CVB3, cardiomyocytes exhibit aberrant antigens that are subsequently identified by T cells and B cells, ultimately resulting in the necrosis of cardiomyocytes.^195^ Released by impaired cells, DAMPs are able to bind to PRRs on monocytes, stimulating the secretion of chemokines, such as CCL2 and MIF-α,^196^ which in turn initiate the recruitment of monocytes and the activation of macrophages, thus accelerating early inflammatory responses and later myocardial remodeling.^195,197–199^ (Fig. 3b) Ly6C^high^ M1 macrophages are dominant in the early inflammatory response and contribute to the removal of viruses and necrotic cells. In contrast, Ly6C^low^ M2 macrophages predominate during later myocardial remodeling, attenuating the inflammatory response and promoting myocardial fibrosis.^200,201^ After viral invasion into the myocardium, cardiac infiltrating macrophages release significant amounts of cytokines and MMPs in response to the infection, primarily including IL-1β, IL-6, TNF-α, and MMP-9.^202,203^ In this process, by activating macrophage NLRP3 inflammasome, CVB3 induces the production of IL-1β, thereby facilitating myocardial injury.^204^ Meanwhile, CVB3 upregulates miR-223^205^ and miR-19b-3p^206^ in cardiac infiltrating macrophages, which activate the NF-κB pathway and trigger the release of the inflammatory factor TNF-α, leading to myocardial injury. Regarding MMP-9 secreted by macrophages, not only does it contribute to ECM hydrolysis and the blockade of viral transmission, but it also lowers the chemotactic activity and diminishes the invasion of inflammatory cells by influencing the expression of IFN-β, IFN-γ, IL-6, and MIP-1, subsequently decreasing the inflammatory response and fibrosis in viral-induced myocarditis.^207^ In terms of promoting later fibrosis, the virus induces macrophages to secrete IL-1, which may bring about an elevation in circulating levels of IL-6, thereby facilitating myocardial fibrosis.^208^ Concurrently, macrophages are also prompted by virus to express iNOS and synthesize excess nitric oxide (NO), amplifying the activation of p44/42 MAPK in CFs and augmenting the expression of CTGF, whose functions include stimulating the proliferation of CFs and enhancing collagen secretion.^209^ Furthermore, the initiation of vitamin D signaling in macrophages results in the upregulation of pERK and the secretion of OPN, which then acts on fibroblasts to enhance the expression of type I collagen through the OPN-ERK-Elk1 pathway and the PI3K cascade, ultimately resulting in fibrosis.^210^

#### Dilated cardiomyopathy

DCM is a primary cardiomyopathy characterized by left ventricular or biventricular dilation, accompanied by decreased ventricular systolic function.^211^ The possible causes of DCM include heredity, poisoning, infection, endocrine, metabolic disorders, and other factors. These factors can induce DNA damage and ROS production, resulting in mitochondrial dysfunction, cellular vacuolation, myocardial apoptosis, and interstitial fibrosis.^211^ Infusing doxorubicin (DOX) and truncating titin variants are the most commonly used models for constructing DCM. Under inflammation induced by damage factors such as DOX, pro-inflammatory macrophages derived from peripheral blood monocytes are the main subset of macrophages in DCM.^212^ ROS is a critical factor in causing damage in DCM **(**Fig. 3c**)**, and its production partly depends on the activation of TLR4 pathway.^213^ Furthermore, TLR4 has been reported to be associated with fibrosis.^214^ Shimazu et al. discovered that myeloid differentiation factor 2 (MD-2), synthesized by monocytes, was essential for TLR4 activation in DCM.^215^ MD-2 directly acts on monocytes and ECs through TLR4/NF-κB pathway to stimulate the synthesis of chemokines and pro-inflammatory cytokines, which could facilitate monocyte recruitment and macrophage activation.^216,217^ Moreover, the NLRP3 inflammasome, synthesized by recruited macrophages in DCM, facilitates the cleavage of apoptosis-associated speck-like protein containing a CARD (ASC), caspase-1, IL-1β, IL-18, and gasdermin-D (GSDMD) into active states, which promote inflammation, cardiomyocyte pyroptosis and myocardial fibrosis.^218,219^ In genetic DCM, recruited macrophages are also the main source of OPN.^220^ Infiltrating macrophages may promote the secretion of galactin-3 via OPN, which will facilitate fibrosis.^220^

Despite not being the dominant subpopulation in DCM, resident macrophages are beneficial to mitigate fibrosis and adverse cardiac remodeling.^212,221^ As for the proliferation of resident macrophages, cardiomyocytes activate resident macrophages by transient receptor potential vanilloid 4-dependent pathways.^183^ Additionally, DOX can induce the production of lipid peroxidation products, which produce class A1 scavenger receptor (SR-A1) ligands. These ligands act on SR-A1 on the surface of macrophages and activate the downstream c-Myc signaling pathway to promote resident macrophage proliferation.^212^ In terms of regulating fibrosis, resident macrophages are capable of actively ingesting dysfunctional mitochondria and other cellular debris released from cardiomyocytes through the phagocytic receptor Mertk, thereby improving myocardial metabolism and inhibiting fibrosis.^222^ In addition, CTGF, which serves as a downstream mediator of the TGF-β pathway as well as boosts the proliferation of fibroblasts and the production of ECM, is upregulated in DCM.^223^ The secretion of insulin-like growth factor 1 (IGF-1) by resident macrophages can effectively suppress fibrosis and enhance cardiac function by inhibiting the production of CTGF.^146,223^ Under the M1/M2 paradigm, M2-like macrophages can transfer mitochondria to injured cardiomyocytes via exosome or extracellular vesicle dependent pathways, thereby inhibiting oxidative stress. This transfer of mitochondria may explain why the adoptive transfer of M2 macrophages can alleviate myocardial fibrosis.^32,33^

#### Diabetic cardiomyopathy

Diabetic cardiomyopathy is defined as myocardial structural and functional abnormalities in diabetics, with metabolic disorders and myocardial fibrosis being prominent features.^224^ In general, pathophysiological processes related to diabetic cardiomyopathy, such as glucose abnormality, deposition of advanced glycation end products (AGEs), release of adipokines, activation of RAAS, microvascular dysfunction, and oxidative stress, collectively contribute to the infiltration of macrophages into the cardiac interstitial space. Efferocytosis of macrophages, as well as the secreted bioactive mediators TNF-α and resistin, play crucial roles in the metabolic disorders of diabetic cardiomyopathy, especially the hyperglycemic state and the accumulation of harmful substances. Macrophages exposed to high glucose reduce the expression of miR-126, resulting in a corresponding increase in the expression of A distegrinin and metalloprotease 9 (ADAM9). ADAM9 can enhance high glucose-induced cleavage of MerTK, leading to shedding of soluble Mer (sMER) and loss of MerTK function,^225^ which brings about adverse consequences such as defective elimination of abnormal mitochondria in myocardial tissue, obstruction of clearance of apoptotic cardiomyocytes, extracellular accumulation of metabolic wastes, ultimately causing imbalance of cardiometabolic balance and ventricular dysfunction.^222,225^ In diabetic cardiomyopathy, macrophages secrete large amounts of TNF-α, which can significantly reduce the content of cellular glucose transporter 4 (GLUT4) and the tyrosine phosphorylation level of insulin receptor substrate 1 (IRS1), causing impairment of glucose uptake by heart tissue cells.^226–228^ Furthermore, pro-inflammatory cytokines represented by TNF significantly increase the expression of resistin (an adipokine that contributes to insulin resistance) in macrophages, which further helps maintain a high glucose state.^229,230^ It is worth noting that resistin can also promote the expression of inflammatory cytokines, which means that they promote the production of each other, thus forming a vicious loop.^231^

Macrophages further interact with fibroblasts, ultimately leading to interstitial and perivascular fibrosis^232,233^ (Fig. 3d). Hyperglycemia, one of the main characteristics of diabetic cardiomyopathy, triggers an inflammatory response in macrophages, contributing to the development of myocardial fibrosis.^234,235^ Dectin-1, a PRR primarily expressed on macrophages, plays a crucial role in mediating inflammatory responses in innate immunity and is significantly upregulated in the heart tissue of diabetic mice.^236^ Under the influence of high glucose, Dectin-1 favors the transformation of macrophages into an inflammatory phenotype by stimulating the activation of the spleen tyrosine kinase (Syk)/NF-κB pathway.^236^ High glucose levels stimulate macrophage expression of galectin-3, leading to increased NF-κB p65 activation. This activation, in turn, induces macrophage infiltration into the heart and promotes M1 macrophage polarization.^237^ Meanwhile, abnormal glycemic variability (changes in blood glucose over time) in diabetics promotes M1 macrophage polarization through sodium-glucose cotransporter 1 (SGLT1).^238^ These inflammatory macrophages secrete inflammatory cytokines, including TNF-α, IL-1β, IL-6, etc., which act on CFs and promote the occurrence of myocardial fibrosis. Notably, substance P (SP) can promote the transformation of macrophages into M2 phenotype, playing an important role in regulating ECM remodeling. However, SP is significantly decreased in diabetic hearts, resulting in a greatly elevated proportion of M1 macrophages under high glucose conditions.^239^ In addition to inducing M1 macrophage polarization, hyperglycemia can also facilitate the secretion of pro-fibrotic factors by macrophages, which directly target CFs. Macrophage-derived exosome-associated HuR, an RNA-binding protein, is secreted more under the induction of hyperglycemia and can directly act on fibroblasts to upregulate the expression of fibrosis-related genes.^240^ The development of diabetic cardiomyopathy is often accompanied by the activation of RAAS,^241^ which promotes macrophage to infiltrate into the myocardium and secrete OPN. As an important pro-fibrotic substance, OPN promotes CF attachment to the ECM, and CF growth and ECM production.^242^ Adiponectin (APN) is an adipokine with anti-inflammatory function that can inhibit the Ang-II-induced inflammatory response by activating macrophage autophagy, thereby reducing the degree of myocardial fibrosis.^243^ However, its levels are significantly reduced in diabetic hearts, increasing myocardial fibrosis.^243,244^

#### Cardiac aging

Cardiac aging is characterized by pathological changes in the heart, including hypertrophy, systolic and diastolic dysfunction, lipid deposition, and fibrosis, which culminates in HF. These changes are influenced by factors that occur with age, such as telomere shortening, oxidative stress, metabolic dysfunction, and epigenetic changes^245,246^ (Fig. 3e). As an individual ages, cardiac resident macrophages are gradually replaced by monocyte-derived CCR2^+^ macrophages.^247,248^ This transition is primarily attributed to the accumulation of ROS resulting from dysfunctional mitochondria caused by impaired autophagy function in the aging heart.^249^ ROS activates the Ras-Erk pathway in fibroblasts to promote the high expression of MCP-1, which in turn prompts monocytes infiltration and polarization into M2a macrophages.^250–252^ Additionally, ROS induces vascular ECs to express high levels of the adhesion molecule vascular cell adhesion molecule 1 (VCAM-1), which further facilitates monocyte infiltration into the heart.^253^ The accumulation of macrophages in the aging heart gives rise to a significant secretion of MMP-9, which plays a crucial role in the progression of aging-related interstitial fibrosis. MMP-9 can directly cleave and activate latent TGF-β in the ECM, leading to the expression of pro-fibrotic periostin (POSTN) and CTGF.^254^ Excessive MMP-9 levels also diminish the expression of angiogenesis-related genes, such as integrin β3 and platelet/endothelial cell adhesion molecule 1, resulting in insufficient angiogenesis and an imbalanced oxygen supply to cardiac tissue.^255^ This imbalance sets off inflammatory responses that are critical to subsequent fibrosis.^256,257^ Furthermore, MMP-9 plays a role in regulating macrophage subtypes by promoting their conversion to an inflammatory M1 phenotype.^258^ It is worth mentioning that SPARC produced by fibroblasts, which increases alongside MMP-9, also fosters M1 macrophage polarization.^259^ These factors contribute to the chronic inflammatory state of the aging heart, leading to the release of fibrotic cytokines and growth factors and ultimately triggering the accumulation of collagen in the ECM.^260^

## Heterogeneity and regulatory mechanisms of vascular macrophages

### Atherosclerosis

**Fig. 4:**
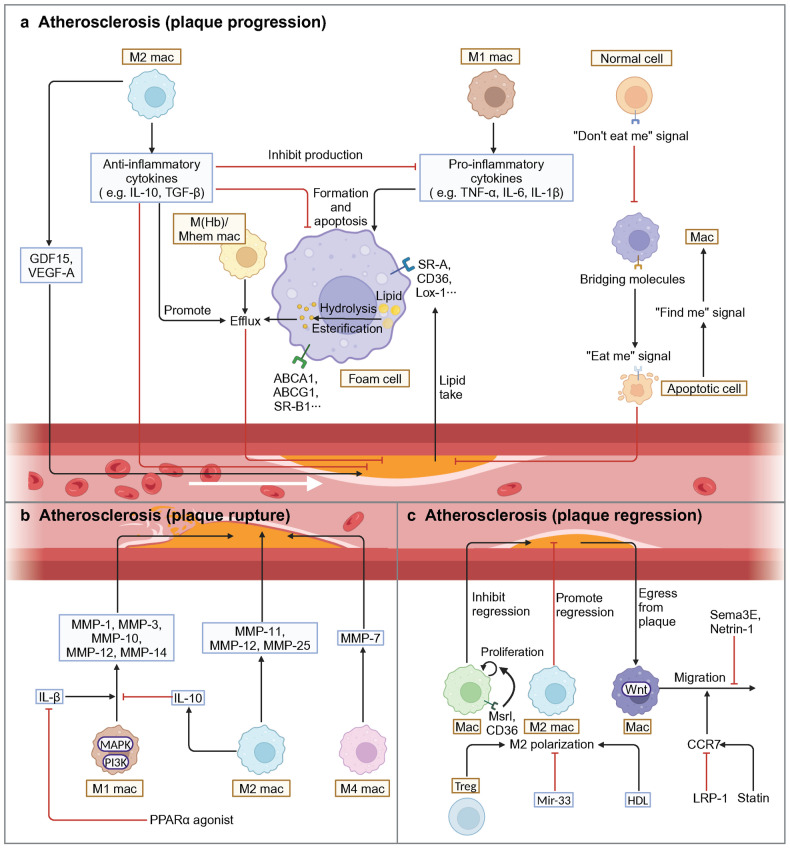
Regulatory mechanisms of macrophages in AS. **a** Foam cells not only bind and uptake circulating lipids to promote plaque progression, but also facilitate cholesterol efflux to prevent plaque progression. M1 macrophages promote foam cell formation mainly by releasing pro-inflammatory cytokines (e.g., TNF, IL-6, IL-1). M2 macrophages secrete anti-inflammatory cytokines (e.g., IL-10 and TGF-β), which inhibit pro-inflammatory cytokines release and foam cell formation, plaque formation, and promote cholesterol efflux. However, M2 macrophages can also promote plaque formation by releasing GDF15 and VEGF-A. Besides, M(Hb) and Mhem macrophages can also mediate cholesterol efflux. Apoptotic cells, which promote plaque formation, release a “Find-me” signal to attract macrophages, and then the “Eat-me” signal on the surface of apoptotic cells combines with the “bridging molecules” signal on the surface of macrophages to initiate the phagocytosis process, while normal cells avoid being phagocytosed by macrophages through the “Don’t eat-me” signal. **b** Macrophage-derived MMPs thin the fibrous cap by directly degrading ECM proteins such as collagen and elastin, causing plaque rupture. M2 macrophages can inhibit the production of MMPs by M1 macrophages through the secretion of IL-10. Clinical PPARα agonists have been found to suppress the production of MMP-12 induced by IL-1β. **c**. During plague regression, the Wnt signaling pathway in plaque macrophages is activated to promote macrophage migration, and Sema3E and netrin-1 inhibit this process. Statins and LRP-1 deficiency promote the regression by activating the CCR7-dependent migration pathway in macrophages. However, scavenger receptors Msr1 and CD36 inhibit plaque regression by promoting macrophage proliferation. M2 macrophages are the main promoters of plaque regression. Tregs and HDL could promote M2 polarization, while miR-33 inhibits M2 polarization. (Created with BioRender.com)

AS is a chronic inflammatory response driven by lipids, and the pathological basis is the accumulation of OxLDL in the arterial intima^261^ (Fig. 4). As a key mediator of inflammatory response, macrophages are involved in all stages of AS development, including plaque germination, calcification, rupture, and regression.^8^

#### Plaque progression

Plaque progression refers to pathological processes such as leukocyte infiltration, lipid accumulation, necrotic core expansion, and fibrous cap formation in AS plaques.^262,263^ AS plaques locally produce chemokines, such as CCL2, CCL5, CX3CL1 and CXCL12, which bind to receptors on monocytes to promote their migration from the blood into tissues. CCR2, CCR5, and CX3CR1 are important receptors on the surface of monocytes that can affect the outcome of AS plaques. After migration to the corresponding site, monocytes also need to enter the vessel wall through transepithelial migration, which is mainly achieved by the adhesion molecules on monocytes (e.g., LFA1, PSGL1) acting on ECs.^264^ The increased number of plaque macrophages depends on increased myelopoiesis of monocytes,^265,266^ induction of chemokines (like CCR2),^267,268^ increased expression of intra-plaque migration inhibitory molecules (like netrin-1)^269^ and macrophage self-proliferation.^270^

Macrophages affect the progression and complication of AS and the formation of rupture-prone plaques by mediating inflammation, lipid metabolism, and efferocytosis (Fig. 4a). M1 macrophages dominate in progressive plaques, mainly by releasing pro-inflammatory cytokines, including IL-1, IL-6, TNF, etc., promoting chronic inflammation of plaques and causing plaque progression and instability.^8,263,271–274^ The pro-inflammatory NLRP3 inflammasome/IL-1 axis has been most extensively studied, which promotes thrombosis and plaque progression through mechanisms such as myeloid cell recruitment, EC activation and angiogenesis.^275–279^ IL-6 promotes the development of AS by inducing vascular smooth muscle cells (VSMC) proliferation, activating ECs, promoting thrombosis, and promoting lipid accumulation in macrophages.^280,281^ TNF is associated with endothelial dysfunction and can promote ROS production, reduce NO bioavailability and increase endothelial permeability.^274,282^ Absence of TNF can attenuate the development of AS disease,^283–285^ but some studies have also produced conflicting results.^286^ Other pro-inflammatory cytokines produced by plaque macrophages are generally considered to promote plaque progression and increase the death risk in patients, such as IL-8,^287,288^ IL-12,^289,290^ and IL-18.^291–293^ M2 macrophages can secrete anti-inflammatory cytokines, including IL-10 and TGF-β, which help to terminate inflammation and inhibit the formation of necrotic core.^47–49^ IL-10 has anti-inflammatory properties and has a protective effect on AS,^294,295^ which may be achieved by inhibiting the release of pro-inflammatory factors,^296^ MMP-9 and apoptosis-inducing substances like caspase-3.^297^ In addition, by upregulating the transporters ATP-binding cassette transporter A1 (ABCA1) and ABCG1, IL-10 also increases cholesterol efflux and disposal of harmful lipoproteins by macrophages.^297,298^ Another anti-inflammatory cytokine, TGF-β, is generally believed to prevent AS and stabilize plaques by inhibiting inflammation, promoting cholesterol efflux from macrophages, and promoting collagen secretion.^299–301^ However, the growth differentiation factor (GDF) 15 of the TGF-β family seems to have a role in promoting the progression of AS.^302,303^ It is worth noting that not all M2 macrophages will contribute to the regression of AS. CD163^+^ M2 macrophages promote angiogenesis, vascular permeability, and leukocyte infiltration through the CD163/HIF1α/VEGF-A pathway, thereby promoting AS progression.^304,305^ OxLDL inhibits the expression of Krüppel-like factor (KLF) 2 in M2 macrophages, thus enhancing the production of pro-inflammatory cytokines such as IL-6 and MCP-1.^306^ This suggests that we need to distinguish M2 macrophages formed by different stimuli, or use other more sophisticated classifications, and be aware of the transformation of macrophage functional phenotypes under different stimuli and environments.

Macrophages in atherosclerotic plaques uptake apolipoprotein B-containing lipoproteins (apoB-LPs) to form lipid-dense cells called foam cells.^52^ After formation, foam cells activate the endoplasmic reticulum stress and apoptosis pathways and release MMPs, which are involved in the process of plaque necrotic core expansion and have pro-atherosclerotic function.^307,308^ Compared with non-foamy macrophages, foamy macrophages express few inflammatory genes but more lipid processing genes.^308^ Cholesterol metabolism in macrophages includes three stages: uptake, esterification, and efflux, of which the uptake and efflux stages have received more attention. The uptake process is the main step of the intracellular accumulation of modified LDL and the formation of fatty streaks. However, if the function of cholesterol efflux is effective, the formation of foam cells and the development of foam cells into apoptotic cells will be inhibited.^297^ SR is a class of receptors on the cell membrane of macrophages and other cell types, which is involved in the removal of many foreign substances and wastes through extensive ligand specificity. Macrophages can bind and uptake circulating lipids through several SRs, such as SR-A,^309,310^ CD36^309–312^ and lectin-like oxidized LDL receptor-1(LOX-1).^313–315^ Sustained activation of SR-mediated uptake processes leads to lipid accumulation and cell necrosis, which facilitates the progression of plaques to more advanced necrotic lesions. Ox-LDL binds to CD36 and triggers the TLR4/TLR6 complex, which initiates sterile inflammation.^316^ The combined elimination of SR-A and CD36 results in the downregulation of inflammatory genes such as Il-1α and Ccl2, and a significant reduction in macrophage apoptosis and plaque necrosis.^309^ LOX-1 promotes inflammatory response and AS progression by activating the NF-κB and MAPK pathways.^314^ After cellular uptake, the modified lipoproteins are carried to intracellular lysosomes for hydrolysis and esterification. Macrophages facilitate cholesterol and phospholipid efflux through multiple transporters, such as ABCA1, ABCG1 and SR-B1, which prevent excessive accumulation of intracellular cholesterol and formation of foam cells.^317–321^ The effects of ABCG1^322^ and SR-B1^323^ on AS may depend on the stage of AS development, related to the functional diversity of these molecules. Deficiency of ABCG1 leads to the accumulation of cholesterol in the early stage of AS, resulting in an enlarged plaque lesion area.^322^ However, in the late stages of AS, cholesterol accumulation caused by ABCG1 deficiency leads to increased macrophage apoptosis, which reduces the susceptibility to AS and delays the progression of lesions.^322^ The dual role of SR-B1 in cholesterol homeostasis may be due to the fact that SR-B1 mediates both the uptake of cholesterol-rich lipoproteins and the efflux of cholesterol to high-density lipoprotein (HDL).^323^ Except for foam cells, M(Hb) macrophages and Mhem macrophages are also involved in lipid metabolism. Compared with foam cells, M(Hb) and Mhem macrophages express high levels of liver X receptor (LXR)-α involved in cholesterol efflux and low levels of SR involved in lipid uptake, thereby promoting cholesterol efflux and preventing foam cell formation.^60,324^ Besides, since iron levels in macrophages may drive cholesterol efflux, manipulating iron levels and iron metabolism-related substances like hepcidin in macrophages can inhibit the generation of foam cells and the development of AS.^325,326^

Efferocytosis is the process by which macrophages eliminate apoptotic cells, thereby limiting secondary necrosis caused by apoptotic cells^327–329^ and terminating the inflammatory response,^271,330^ which is conducive to preventing the progression of AS. Apoptotic cells release a “Find-me” signal to attract macrophages, and then the “Eat-me” signal (such as phosphatidylserine and intercellular adhesion molecule 3 (ICAM-3)) on the surface of apoptotic cells combines with the “bridging molecules” signal (like mammary-derived growth factor 8 (MFGE8)) on the surface of macrophages to initiate the phagocytosis process. Living cells avoid being phagocytosed by macrophages through the “Don’t eat-me” signal, such as CD47 and CD31. SR-B1 on the surface of macrophages mediates efferocytosis and reduces atherosclerotic lesion necrosis through intracellular Src/PI3K/Rac1 signaling.^331^ M2 macrophages in plaques show higher phagocytosis than M1 macrophages, which is due to the involvement of highly expressed opsonins and receptors involved in phagocytosis, such as PPARγ^332^ and Mertk.^333,334^ In the early stage of AS, macrophages exhibit a capacity to respond to apoptosis, thereby mitigating the expansion of the necrotic core within atherosclerotic plaques^328^ As plague progresses, macrophage efferocytosis within plaques is impaired, leading to chronic and unresolved inflammation and enhanced macrophage apoptosis in advanced plaques, ultimately promoting the formation of a necrotic core.^335–337^ Impaired efferocytosis in advanced plaques is mainly caused by lipid competition for recognition receptors,^338,339^ downregulation of “bridging molecule” signals, upregulation of “Don’t eat me” signals,^327,340^ and impairment to mitochondrial fission.^341^ CD47 binds to inhibitory signal regulatory protein α (SIRPα) on macrophages to induce the “Don’t eat-me” signal. CD47 blocking antibodies or SIRPα deletion improve efferocytosis in plaques, attenuate oxidized LDL-induced inflammation and induce M2 macrophage polarization, thereby reducing the formation of necrotic core.^342–344^

Arterial calcification is caused by the crystallization of calcium and phosphate in the form of hydroxyapatite, which can accumulate in the ECM of the artery wall. The degree of plaque calcification is also a measure of plaque stability.^345^ The inability of microcalcification formed by M1 macrophages to form stable structures is associated with an increased risk of plaque rupture.^346,347^ However, macrocalcification formed by M2 macrophages can stabilize AS plaques.^348^ M1 macrophages induce osteogenic transdifferentiation of VSMCs and further mineralization of plaque lesions mainly by secreting pro-inflammatory cytokines (such as IL-1β and TNF-α).^349–351^ Anti-inflammatory cytokines (like IL-10) secreted by M2 macrophages may be beneficial to osteoblastic differentiation of VSMCs and plaque macrocalcification.^348^ In addition, OSM secreted by plaque macrophages induces osteoblastic differentiation of VSMCs and M2 macrophage polarization through the Janus Kinase 3 (JAK3)/STAT3 pathway, thereby promoting plaque macrocalcification and stability.^352^

#### Plaque rupture

Rupture-prone plaques contain a large necrotic core and a thin fibrous cap, and are also characterized by high MMP activity, ECM proteolysis, VSMC dedifferentiation, impaired exocytosis and chronic inflammation^353^ (Fig. 4b). Among them, macrophage-derived MMPs thin the fibrous cap by directly degrading ECM proteins such as collagen and elastin,^53^^,^^354–356^ so MMP-1, MMP-8, and MMP-12, which belong to collagenase, have a greater impact on plaque stability.^357^ Newly recruited monocytes may upregulate a broad spectrum of MMPs through a prostaglandin (PG)-dependent pathway.^358^ Different macrophages secrete different MMPs to participate in plaque rupture. M1 macrophages mainly release MMP-1, MMP-3, MMP-10 and other MMPs, while M2 macrophages reduce MMP-2 and increase MMP-11, MMP-12, MMP-25 and other MMPs.^359^ And M4 can participate in fibrous cap degradation and plaque rupture by producing MMP-7.^360^ Clinically, PPARα agonists are used to lower lipids for the treatment of AS. PPARα agonists have also been found to inhibit IL-1β-induced MMP-12 production, thereby preventing inflammation and plaque rupture.^361^

#### Plague regression

As LDL-cholesterol in circulating blood continues to decrease, plaque regression may occur. During the regression process, plaque composition can change significantly from that of progressive plaques, with increased fibrotic cap thickness,^362^ decreased macrophage content, and M2 macrophage polarization^47,363–365^ (Fig. 4c). At present, the mechanisms underlying plaque regression are relatively less studied than those driving plaque progression, and mainly rely on a cholesterol-free diet or the use of cholesterol-metabolizing drugs (e.g., statins and ezetimibe).^366–368^ The reduction in the number of plaque macrophages mainly depends on the inhibition of local proliferation^369,370^ and the efflux of macrophages from the site of inflammation.^371^ One study showed that Msr1 and CD36,involved in the uptake of modified lipoproteins, are mediators of plaque macrophage proliferation.^369^ Statins and low-density lipoprotein receptor–related protein 1 (LRP-1) deficiency promote the regression of AS by activating the CCR7-dependent migration pathway in macrophages.^372–374^ During regression, the Wnt signaling pathway in plaque macrophages is activated to promote macrophage migration.^375^ At the same time, the classical Wnt/β-catenin signaling regulates the STAT pathway in macrophages to terminate the elevated inflammatory response and prevent AS.^376^ Sema3E^377^ and netrin-1^269^ are upregulated in macrophages in advanced plaques, which serve as negative regulators of macrophage migration, promoting macrophage retention and chronic inflammation, and targeted inhibition of negative regulators facilitates plaque regression. Notably, inhibition of monocyte recruitment was found to be critical for plaque macrophage regression in a model of plaque regression.^378^ In regression plaques, macrophages exhibit downregulation of adhesion-related genes (e.g., cadherin, vinculin) and upregulation of movement-related genes (e.g., actin and myosin) and M2 phenotype-related genes (e.g., arginase I and CD163).^379^ Tregs are essential for macrophage efflux, M2 polarization and pro-catabolic functions in regressing plaques, including clearance of apoptotic cells and cellular debris and production of specialized pro-lipolytic mediators.^380^ The antagonism of miR-33, a microRNA that is elevated in macrophages in progressive lesions, promotes macrophages tilt toward the M2 state and causes plaque regression.^381,382^ During plaque regression, the increase in the concentration of functional HDL particles is an important contributor to plaque regression. HDL can mediate cholesterol efflux and induce M2 polarization,^383,384^ which is dependent on the STAT6 pathway^385^ and the expression of activating transcription factor 3 (ATF3).^386^

### Aneurysm

**Fig. 5:**
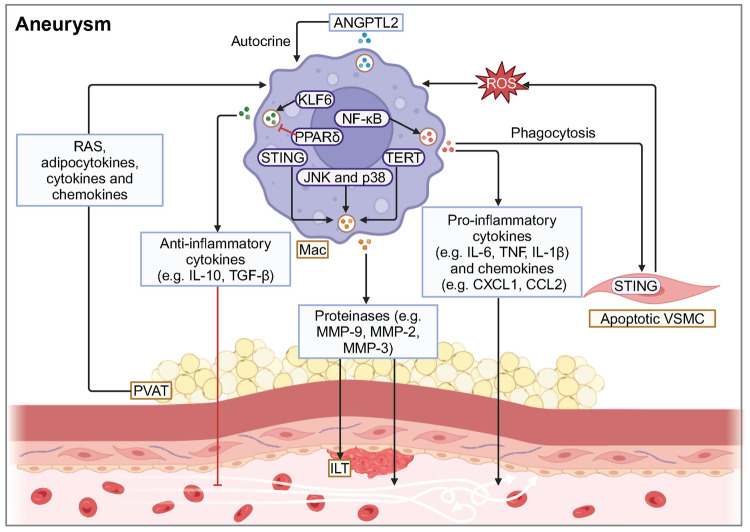
Regulatory mechanisms of macrophages in aneurysm. Macrophages mainly regulate inflammatory response, ECM remodeling and VSMC apoptosis in AA. Macrophages clear apoptotic VSMCs through phagocytosis and produce large amounts of ROS, which further activate macrophages. Macrophages can also secrete pro-inflammatory cytokines (such as IL-6, TNF, IL-1β), chemokines (such as CXCL1 and CCL2) and ANGPTL2 to promote the development of inflammation. On the contrary, macrophages also secrete anti-inflammatory factors such as IL-10 and TGF-β by upregulating the transcription factor KLF6 or downregulating the activation of PPARδ. In addition, macrophages release proteinases such as MMP-9, MMP-2, and MMP-3 by activating the STING, TERT, JNK and p38 pathways, leading to aortic wall bleeding and rupture. In turn, macrophage recruitment, accumulation, proliferation, and activation are modulated by microenvironmental conditions (such as hemodynamics, circumferential stress, PVAT and ILT). (Created with BioRender.com)

Aneurysms generally occur in the aorta, and the main pathological characteristics of aorta aneurysms (AA) are smooth muscle cell (SMC) apoptosis, inflammatory response and matrix degradation.^387^ Macrophages play an important role in all stages of AA development, and are affected by the microenvironment such as hemodynamics, changes in circumferential stress, perivascular adipose tissue (PVAT) and intraluminal thrombus (ILT) (Fig. 5). M1 macrophages are involved in the development of AA mainly by secreting inflammatory factors and MMPs, promoting ECM destruction and VSMCs apoptosis.^46,388^ However, M2 macrophages are involved in vascular repair mainly by inhibiting inflammation.^46,388,389^ Since it is crucial to inhibit further development and rupture of AA, studies have mostly focused on early M1 macrophages.

When an artery is injured, monocytes are recruited to the injury site by chemokines such as CCR2 and CX3CR1, and differentiate into macrophages.^390–392^ Inflammatory response is one of the main characteristics of AA, and macrophages play an important role in regulating inflammation. M1 macrophages promote inflammation by releasing ROS, pro-inflammatory cytokines, and chemokines. When M1 macrophages clear early cell debris through phagocytosis, they produce large amounts of ROS, which together with ROS derived from ECs, VSMC and other immune cells in the aortic wall further activate macrophages, thus continuously enhancing this cycle.^393–397^ M1 macrophages can also secrete pro-inflammatory cytokines (such as IL-6, TNF, IL-1β, etc.) to promote the development of inflammation.^388,398–400^ Recent studies have found that S-Nitrosylation of Septin2 and adenosine deaminase acting on RNA (ADAR1) in macrophages in AA can promote the activation of the NF-κB signaling pathway, which in turn activates NLRP3 inflammasome, resulting in the release of IL-1 and enhanced degradation of ECM.^401–403^ Activation of NLRP3-caspase-1 inflammasome is also associated with the degradation of contractile proteins.^404^ Infiltrating macrophages can also highly express angiopoietin-related protein 2 (ANGPTL2), which induces macrophages to further release pro-inflammatory factors such as TNF-α, IL-1β, and IL-6 and MMPs in an autocrine manner.^405^ In addition to inflammatory factors, M1 macrophages promote the recruitment of inflammatory cells by producing chemokines such as CXCL1 and CCL2, forming a positive feedback that continuously promotes chronic inflammation.^406–408^ Among them, CXCL1 recruits neutrophils which secrete IL-6, and the increase in IL-6 levels in turn promotes the differentiation of monocytes into macrophages which secrete CCL2, thus recruiting more monocytes into the aneurysmal artery wall.^395,409,410^ In contrast to M1 macrophages, M2 macrophages promote vascular reconstruction and repair by secreting anti-inflammatory factors such as IL-10 and TGF-β, inhibiting the production of inflammatory factors and MMPs, clearing hemoglobin, and regulating oxidative stress,^46,406,411,412^ which may be achieved by upregulating the transcription factor KLF6 or downregulating the activation of PPARδ.^413,414^ Nevertheless, it has been found that the deficiency of IL-12p40 promotes the development of abdominal aortic aneurysms by promoting the recruitment of M2 macrophages.^415^ Therefore, M2 macrophages are not exclusively beneficial to aneurysms.

In addition to inflammation, ECM degradation is also one of the main features of aneurysms. Macrophages release proteinases such as MMP-9, MMP-2, and MMP-3 to degrade the ECM, leading to aortic wall bleeding and rupture.^395^ Among them, MMP-9 may play a more important role in AA due to its highest content.^416,417^ Luo et al. found that SMC damage and subsequent DNA release into the cytoplasm activated the STING-TBK1-IRF3 pathway, promoting SMC apoptosis and necrosis.^418^ Macrophages phagocytose DNA released by damaged SMCs and activate stimulator of interferon genes (STING) and its target protein IRF3, which enters the nucleus and binds to the MMP-9 promoter to induce MMP-9 expression.^418^ MMP-2 is also the primary MMPs during the early stages of AA formation, leading to the initial breakdown of elastic tissue.^419,420^ Telomerase reverse transcriptase (TERT) in bone marrow-derived macrophages promotes MMP-2 expression.^421^ Besides, there are a large number of exosomes in the adventitia of aneurysmal arteries, mainly from macrophages, which can induce the expression of MMP-2 in VSMCs by activating the JNK and p38 pathways.^422^

The microenvironment of AA, including hemodynamics, changes in circumferential stress, PVAT and ILT, can also influence macrophage action. Increased aortic blood flow and wall shear stress can promote macrophage apoptosis, induce the expression of antioxidant genes such as HO-1 in macrophages, and reduce ROS production.^423,424^ However, lower shear stress can induce inflammatory responses by promoting macrophage infiltration.^425^ PVAT induces endothelial dysfunction and macrophage infiltration by secreting RAS components, adipocytokines, cytokines, and chemokines like CCL2, promoting inflammation and aortic dilation.^426–429^ Adverse remodeling following ECM degradation can lead to the formation of ILT.^395^ ILT can form an inflammatory microenvironment containing cytokines, proteinases and ROS. At the same time, its growth competes for oxygen and nutrients, causing local hypoxia in the aortic wall, which is related to the activation of inflammatory macrophages, increased inflammation levels, degradation of elastin in the arterial wall and the decrease of SMCs, thereby damaging the arterial wall.^388,426,430^

## New perspectives brought by single-cell technologies

Single-cell technologies, particularly scRNA-seq, allow resolution of gene expression at the single-cell level to reveal cellular heterogeneity. Compared with lower resolution sequencing technologies such as RNA-seq and bulk RNA-seq, scRNA-seq can perform accurate and unbiased cell clustering, discover rare cell subsets, and provide transcriptome profiles of cell subsets rather than just several markers through a series of algorithms.^431,432^ In addition, scRNA-seq can also perform multi-dimensional data mining, including revealing the differentiation trajectory of key cell populations, the interaction between cell populations in specific physiological and pathological states, and the identification of key transcription factors.^431^ Thus, scRNA-seq can provide new insights into the complex biological process of macrophage-mediated CVD pathogenesis, which includes uncovering into the diversity of macrophages as well as searching for new mechanisms and potential therapeutic targets.^431^Moreover, spatial transcriptomics (ST) can supplement the positional information at the spatial level lost by scRNA-seq, revealing the spatial distribution of macrophages for exploring the real cell interaction mechanism.^433,434^

### Cardiac macrophages

Previously, recruitment macrophages and resident macrophages could be roughly distinguished by CCR2 expression. However, recent scRNA-seq studies have shown that monocyte-derived macrophages can acquire a variety of cell fates, and some of these subsets have low CCR2 expression, suggesting that the use of CCR2 expression to distinguish the origin of macrophages is not precise enough.^13,67,435–437^ Meanwhile, scRNA-seq reveals that the recruited macrophages have low expression of reparative genes such as TIMD4, LYVE1 and folate receptor 2 (FOLR2), and combined with this feature, macrophages of different origins could be better distinguished.^67^ The use of scRNA-seq defines the most dominant resident macrophage subset, namely TLF^+^ (expressing TIMD4 and/or LYVE1 and/or FOLR2) macrophages^18,67,147,435,436^ (Fig. 1b). The renewal of TLF^+^ macrophages is independent of circulating monocytes,^18^ and their transcriptome signatures are mainly functions of maintaining homeostasis, such as endocytosis, cell transport, and angiogenesis.^18,67^ TLF^+^ macrophages have also been found to inhibit fibrosis in MI^67^ and PO.^19,147^ In the context of scRNA-seq applied to AMI, different subsets of recruited macrophages were further divided, and it was found that interferon-stimulated gene (ISG^+^) macrophages and MHC-II^+^ macrophages are important pro-inflammatory subsets in the inflammatory phase, while triggering receptor expressed on myeloid cells 2 (Trem2^+^) macrophages are the major anti-inflammatory subset in the reparative phase (Fig. 1b). ISG^+^ macrophages activate the IRF3-IFN axis by uptake of DNA from infarcted myocardium, which facilitates the production of pro-inflammatory cytokines and chemokines, ultimately worsening cardiac function.^67,435,438,439^ MHC-II^+^ macrophages, another major pro-inflammatory subset, enrich transcripts associated with a pro-inflammatory and pathogenic profile, such as Il1b, Nlrp3, and Tlr2.^67,435,439–441^ Previously, these two pro-inflammatory subsets may have been broadly described as M1 macrophages. Trem2^+^ macrophages predominate in late-infarcted hearts and highly express tissue repair, exocytosis, and anti-inflammatory genes.^434,435,437,440,442^ Injection of soluble Trem2 in mice can inhibit fibrosis and improves infarcted heart function,^434^ and cardioprotective effects of Trem2 have also been found in PO^442^ and sepsis-induced cardiomyopathy.^440^

In terms of exploring new potential mechanisms, recent scRNA-seq studies have found that miR-21, ALKBH5, SPP1, Runx1 and NLRP3 can become new targets for the regulation of macrophage inflammation. MiR-21, an important microRNA driving fibrosis, was found to be essential for the elevation of M1 subsets in PO. Ligand-receptor interaction analysis based on scRNA-seq and in vitro model validation confirmed that M1 macrophages secrete miR-21 in a paracrine manner, which activated the differentiation of CFs into myofibroblasts.^441^ Also in PO, scRNA-seq and lineage tracing revealed that cardiac macrophages derived from circulating monocytes preferentially undergo macrophage-to-myofibroblast transition through the ALKBH5/IL-11/IL-11RA1 axis, resulting in hypertensive myocardial fibrosis and dysfunction in mice.^30^ In the Hulsmans et al. atrial fibrillation mouse model that integrates hypertension, obesity, and mitral valve regurgitation, scRNA-seq suggested that CCR2^+^Trem2^+^ macrophages promoted the progression of fibrosis and atrial fibrillation by secreting SPP1, which was verified in the bone marrow transplantation model.^443^ Ligand-receptor interaction analysis also showed that SPP1 may act on integrins, CD44, and the prostaglandin E2 (PGE2) receptor on fibroblasts to activate the TGF-β pathway.^443^ CCL3^+^ pro-inflammatory macrophages and TNMD^+^ fibroblasts are enriched in the right ventricle of patients with arrhythmogenic right ventricular cardiomyopathy (ARVC). CCL3^+^ pro-inflammatory macrophages strongly interact with fibroblasts via NLRP3, and pharmacological inhibition of CCL3^+^ pro-inflammatory macrophages significantly alleviated RV dilatation and dysfunction in a mouse model of ARVC.^444^ In addition, a single-nucleus RNA sequencing study predicted that downregulation of runt-related transcription factor 1 (RUNX1) transcriptional activity in cardiac macrophages and fibroblasts may promote cardiac recovery in patients with HF by gene regulatory network construction. This possibility was confirmed in subsequent animal experiments.^445^ Utilizing ST, it was found that macrophages were dispersed across the whole heart on day 1 after MI and began to penetrate deep into the infarct area from day 3, and their numbers peaked on days 5 and 7.^434^ Other immune cells, such as B cell and T cell, were always dispersed across the entire mouse heart (not clustered in the infarcted area), which confirmed the importance of macrophages for scar formation.^434^ One study using ST in MI patients observed crosstalk between SPP1^+^ macrophages and neighboring fibroblasts in the infract zone. Moreover, ligand-receptor interaction analysis found that SPP1^+^ macrophages may act on fibroblasts through PDGF-C, PDGF-D, and thrombospondin-1 (THBS1) signaling to affect the progression of fibrosis.^26^ Another study using ST identified monocyte-derived basic helix-loop-helix family member e41 (Bhlhe41^+^) macrophages in the developing infarct zone of MI. By analyzing spatial ligand-receptor interaction and in combination with animal model experiment, the results suggested that Bhlhe41^+^ macrophages could increase the secretion of granulin (GRN) to antagonize the effect of TNF-α on TNFR1, thereby inhibiting myofibroblast activation and limiting the expansion of the infarct zone.^446^ This mechanism was verified by co-culture systems and depletion of Bhlhe41^+^ macrophages in mice. Meanwhile, Bhlhe41^+^ macrophages were found to help limit the expansion of developing infarct area and improve cardiac function.^446^ The combination of ST with single-cell data allows us to have further insight into fibro-myeloid spatial relations in different histomorphological regions (infarcted, border, and remote zones). These results also support the application of ST to explore the spatial distribution patterns and roles of macrophages and other cells in heart diseases.

### Vascular macrophages

scRNA-seq analysis of the diversity of macrophages in mouse and human blood vessels identifies resident macrophages involved in the maintenance of homeostasis, inflammatory macrophages, anti-inflammatory macrophages, and proliferative macrophages in vascular diseases^308,407,447–454^ (Fig. 1b). Resident macrophages are mainly found in the adventitia of healthy and diseased blood vessels.^455^ Resident macrophages are capable of proliferating and resemble an M2-like phenotype, and their transcriptome is characterized by the expression of Lyve-1, FOLR2, F13a1, and Wfdc17, which are involved in signaling pathways related to phagocytosis, intercellular adhesion, chemotaxis, and vascular calcification.^447,449,453,454,456–459^ Inflammatory macrophages in AS and AA are mainly present in the intima and adventitia of the vascular walls, respectively.^455^ Inflammatory macrophages are enriched in M1-related genes, expressing genes encoding pro-inflammatory mediators (including IL-1β, Nlrp3, Tlr2), chemokines (e.g., Cxcl2, Ccl3, Ccl4), and transcription factors (e.g., Cebpb, Egr1).^407,447,448,453,454,457,459^ A special group of IFN-induced macrophages related to inflammation is also found in AS, mainly present in the intima, expressing genes such as Isg15, Irf7, and Ifit1, which promote macrophage recruitment and foam cell formation.^460–462^ Anti-inflammatory macrophages mainly highly express genes related to anti-inflammation, phagocytosis, and proteinase, such as Pf4, Mrc1, Arg1, and Ctsa, promoting anti-inflammation and vascular remodeling.^407,454^ Trem2^+^ macrophages are important anti-inflammatory macrophages that infiltrate diseased blood vessels but are not present in healthy mice. They are lipid-rich and resemble an M2-like phenotype, characterized by the expression of Lgals3, Cd9, Ctsd, and Spp1, and enriched with signaling pathways related to cholesterol metabolism, oxidative phosphorylation, the lysosome, and the proteasome.^447,448,461^ While Trem2^+^ macrophages can regulate LDL levels by removing apoptotic cells and lipids to prevent lipid metabolism disorders and also play an anti-atherosclerotic role by inhibiting inflammation and advanced calcification, they also express some molecules that exacerbate plaque rupture (e.g, Lgals3 and Ctsb).^407,459,463^ Proliferating macrophages represent macrophages that are expanding or renewing, whose transcriptome is characterized by the expression of Mki67, Stmn1, Top2a, and Tuba1b and the enrichment of signaling pathways for cell proliferation.^407,447,454,459,463^

With regard to the comprehension of disease mechanisms, analysis of intercellular interaction based on scRNA-seq shows that macrophages primarily interact with ECs, T cells, and VSMCs.^452^ ECs and macrophages mainly exert adhesion through ICAM1-VCAM1/ITGB2 and ACKR1-CCL8/CXCL1, perform transendothelial migration through SELL–CD44, and participate in angiogenesis through PDGFBR-PDGFB. These functions may be related to the initiation of AS.^448,457^ T cells and macrophages activate each other through VCAN-TLR1/2, CCL5-CCR1/5, and ITGAL-ICAM1 to induce cytotoxicity and antigen presentation and regulate lipid accumulation and foam cell formation by regulating LRP1 ligands on macrophages.^448,451^ For VSMC, CCL5, which is highly expressed by macrophages, interacts with CCR5 on VSMC to drive VSMC proliferation and conversion to the synthetic phenotype, thereby causing vascular remodeling and plaque progression.^451,464^ In addition, scRNA-seq is used to explore the downstream mechanisms of intervention targets for vascular diseases, including netrin-1, miR-33, and CD47/SIRPα, among which netrin-1 is a common target of AS and AA. In AS, silencing of myeloid netrin-1 in mice resulted in downregulation of genes involved in pro-inflammatory responses (S100a8/9) and upregulation of genes involved in lipid metabolism, anti-inflammatory (Il10, Tgfb), and cell migration (Ccr7) in macrophages, thereby promoting resolution of inflammation and reducing plaque burden in the aorta.^465^ In AA, netrin-1 activates the release of MMP-3 in VSMCs, leading to matrix degradation, which promotes the formation of AA. Therefore, a deficiency of netrin-1 can prevent AS and AA.^466^ Anti-miR-33 reduces the proliferation and retention of MHC-II^high^ inflammatory and Trem2^+^ macrophages, decreases the accumulation of vascular lipid, promotes macrophage apoptosis and cytotoxicity clearance, and increases collagen content, thus playing a role in tissue repair and the resolution of inflammation.^467,468^ Interruption of CD47/SIRPα signaling by precision-engineered nanoparticles causes macrophages to downregulate the expression of pro-inflammatory transcription factors (CCL2, CCL7, CCL8, and PF4), upregulate the expression of genes related to inflammation resolution (SOCS3 and Zfp36), and also enrich genes related to phagocytosis and antigen presentation, resulting in the reduction of plaque burden.^469^ Up to now, few studies have been published on ST in vascular diseases.^470,471^ A spatial transcriptional map study found that macrophage-derived MMP-9 was more prominent in the narrowest areas of plaques (unstable) than in the distal areas (stable),^472^ which may help us better understand the characteristics of ruptured plaques.

## Current status of preclinical macrophage targeting strategies

### Inhibition of macrophage recruitment

In cases of inflammation or injury in cardiovascular tissue, recruited macrophages act as the primary inflammatory cells that mediate the balanced regulation of inflammatory immunity and play a central role in the interaction between various cells. Therefore, inhibiting the recruitment of macrophages is a promising therapeutic strategy for CVD.^3^ The most studied CVD is AS. In AS, monocytes aggregate into plaques through chemokine-mediated recruitment,^473^ adhesion molecule-mediated adhesion, and junction adhesion molecule-mediated exudation.^474^ Inhibiting these targets not only prevents the subsequent accumulation and proliferation of macrophages in the plaque but also prevents the instability and rupture of atherosclerotic plaques.^475^ Recruitment of monocytes is primarily mediated by the CCL2-CCR2 axis.^476^ When CCL2 or CCR2 is deficient, macrophage recruitment to the blood vessel wall is reduced in mice, and consequently, atherosclerotic lesion size is also reduced.^477–479^ Conversely, if CCL2 is overexpressed, the number of macrophages and the accumulation of oxidized lipids in mice atherosclerotic plaques are significantly increased, thereby promoting the progression of AS.^480^ One study shows that the combined deletion of CCL2, CX3CR1, and CCR5 significantly reduces macrophage invasion and plaque lesion size compared to deletion alone.^481^ Similarly, the combined loss of CCR2 and CX3CL1 significantly reduces the accumulation of macrophages in the lesions and decreases the instability of atherosclerotic plaques.^482^ These results suggest that targeting multiple chemokines or receptors simultaneously is a potential therapeutic strategy.^476^ For monocyte adhesion, this process is mainly mediated by the binding of VCAM-1 on vascular ECs and very late antigen 4 (VLA-4) integrin on circulating monocytes.^483^ Direct inhibition of VCAM-1 has been shown to prevent monocytes from infiltrating into the subcutaneous space, thereby effectively preventing macrophage maturation and foam cell transformation required for the formation of atherosclerotic lesions.^484^ However, highly specific peptide and antibody therapeutics that selectively inhibit VCAM-1/VLA-4 interactions have recently emerged as a promising adherence-based anti-AS therapy.^485^ During the exudation process, inhibiting the junctional adhesion molecule A (JAM-A) can effectively reduce inflammation and monocyte recruitment to atherosclerotic endothelium, thus decreasing the formation of the AS.^486^ In addition, as an inflammatory cytokine with chemokine-like characteristics, MIF also plays a critical role in the overall macrophage recruitment process.^487,488^ Treatment with MIF antibody in atherosclerotic mice significantly reduces the content of macrophages in the lesion as well as the levels of circulating and local aortic inflammatory mediators, thereby inhibiting the area of plaque development.^489^ In MI, many recent preclinical studies have also focused on targeting the CCL2-CCR2 axis.^473^ Studies have shown that reducing CCR2 expression through CCR2 inhibitors can significantly inhibit monocyte recruitment in the heart, thereby easing the inflammatory cascade and reducing MI size.^490^

### Inhibition of foam cell formation and macrophage survival

Foam cells are prototype cells in atherosclerotic plaques, formed by the excessive accumulation of cholesterol esters by macrophages.^491^ Therefore, inhibiting foam cell formation by targeting critical proteins involved in macrophages cholesterol uptake,^492^ esterification,^493^ and efflux^494^ is one of the important strategies for treating AS. Studies have shown that by silencing SR-A alone, foam cell formation can be significantly reduced, thereby decreasing the occurrence of AS.^310^ However, the role of acetyl coenzyme A acetyltransferase 1 (ACAT-1) in cholesterol esterification in AS is still controversial. Pharmacological inhibition of ACAT-1 has been found to lead to increased foam cell formation in atherosclerotic mouse and rabbit models, which, in turn, facilitates plaque formation.^495^ The overexpression of ACAT-1 also facilitates the accumulation of cholesterol ester and the formation of macrophage-derived foam cells, which increase the occurrence of AS.^496^ Finally, in cholesterol efflux, a related study has found that treatment with PPARα and PPARγ agonists in LDL-receptor deficient mice induces LXRα and LXR-mediated ABCA1 expression, which promotes cholesterol efflux and reduces foam cell formation, thereby inhibiting the development of AS.^497^

The role of macrophage death in CVD is complex. It may either promote tissue repair and remodeling or exacerbate tissue damage and inflammation, depending on the mode and extent of its death.^498,499^ The death modes of macrophages are predominantly apoptosis, regulated necrosis (including necroptosis, pyroptosis, and ferroptosis), and autophagy.^490,499–503^ Apoptosis is an orderly process of cell death that eliminates excess or damaged cells and prevents an inflammatory response.^504^ In contrast to apoptosis, regulated necrosis induces an inflammatory response.^505^ Autophagy is a non-apoptotic form of cell death that prevents inflammation.^499^ Current therapeutic strategies targeting these modes of cell death to regulate macrophage survival have predominantly focused on atherosclerotic disease, with less emphasis on macrophage death modes in the cardiac field. For AS, liposomes containing drugs, such as clodronate, are widely used to induce apoptosis of macrophages because they can be delivered to macrophages through phagocytosis without causing cytotoxicity to non-phagocytes.^506,507^ Studies have shown that the administration of clodronate liposomes (Clo-Lip) inhibits mitochondrial oxygen consumption, leading to macrophage apoptosis and preventing the progression of AS.^508^ However, systemic administration of clodronate-containing liposomes also reduces blood monocytes, which increases the risk of immunosuppression and infection. Notably, when recombinant tumor necrosis factor-related apoptosis-inducing ligand (TRAIL) is administered systemically to mice with diabetes-induced AS, no adverse effects are observed. It is found that TRAIL induces apoptosis of infiltrating macrophages in atherosclerotic plaques but does not induce apoptosis of circulating macrophages, significantly weakening the development of AS.^509^ In terms of targeting macrophage necroptosis, intervention in mice with atherosclerotic lesions using the pharmacological necroptosis inhibitor necrostatin-1 (Nec-1) has been found to prevent further progression of the lesions and reduce markers of plaque instability, known as necrotic core and necrotic cell death.^510^ In addition, the activation of the NLRP3 inflammasome during pyroptosis is required for the formation of AS. Therefore, targeted destruction of the NLRP3 inflammasome significantly protects atherosclerotic mice from the disease and reduces lesion size.^275^ In terms of targeting ferroptosis in macrophages, studies have found that the use of micheliolide (MCL)^511^ or IL-37^512^ can activate the nuclear factor erythroid 2-related factor 2 (NRF2) pathway, thereby inhibiting ferroptosis in macrophages and reducing the progression of AS. Finally, by targeting mechanistic target of rapamycin (mTOR), a critical protein activated by the autophagy pathway, such as a mTOR inhibitor^513^ or silencing mTOR with small interfering RNA,^514,515^ the activation of macrophage autophagy genes can be induced, leading to the clearance of macrophages in plaques and facilitating a stable plaque phenotype. However, in the heart, the immune microenvironment in which macrophages reside is more complex than that in blood vessels. It requires both M1 macrophages to clear dead cells and M2 macrophages to facilitate infarction repair and angiogenesis promptly. Therefore, uniformly targeted strategies for macrophage depletion are often ineffective, hindering wound healing and left ventricular remodeling after myocardial injury.^516,517^ However, it has been found that the absence of the apoptosis inhibitor of macrophage (AIM) selectively reduces the level of M1 macrophages in MI, which decreases the incidence of heart rupture and improves the survival rate.^518^ At present, there are few studies on targeting specific macrophage subsets for cell death in MI, which may be a potential therapeutic strategy to promote post-MI repair.

### Regulation of macrophage function

Macrophages have many functions in the cardiovascular system, such as regulating inflammation and fibrosis, lipid metabolism, efferocytosis, etc. Regulating the function of macrophages is a feasible idea for the treatment of CVD. For the cardiac system, current research is mainly based on ischemic injury models to explore the regulation of macrophage function by cytokines and cell therapy. IL-1, IL-4 and IL-10 are widely studied cytokines that regulate macrophage function. Anti-IL-1β antibody or anakinra (an exogenous recombinant human IL-1Ra) treatment reduces the intensity of inflammation, prevents excessive accumulation of white blood cells, and inhibits cardiomyocyte apoptosis by inhibiting IL-1,^277,519,520^ while systemic infusion or targeted delivery of IL-4 and IL-10 during the inflammatory phase can induce macrophages to produce repair-phase bioactive mediators with anti-inflammatory, pro-angiogenesis, and collagenesis functions.^114,521–523^ Based on this, the infarct size is reduced, the pumping function of the heart is improved, and the degree of adverse fibrosis is reduced after MI.^114,277,519–523^ For cell therapy, the current focus is on the infusion of mesenchymal stem cells (MSCs) and ex vivo reprogrammed macrophages. MSCs regulate the function of macrophages from pro-inflammatory to anti-inflammatory by means of direct intercellular communication or paracrine. They have the advantages of strong immunomodulatory ability, low antigenicity, easy acquisition and easy expansion in vitro, etc., but there are problems such as low survival rate and implantation rate.^524,525^ Reprogramming macrophages in vitro prompts them to produce specific functions such as anti-inflammation, promoting angiogenesis, and preventing myocardial cell apoptosis, which not only allows personalized treatment for patients, but also avoids off-target effects that are prone to occur when regulating macrophage function in vivo.^506^ For example, after infusion of Cardiac Nestin^+^ MSCs, in vitro M-CSF and IL-4 combined treated macrophages or hypoxia-induced in vitro reprogrammed macrophages into MI animal models, it is observed that pathological fibrosis of the heart infarction area or distal end is reduced, microangiogenesis is enhanced, and cardiomyocyte hypertrophy is weakened.^526–528^

For the vascular system, current research is mainly based on AS models to explore the regulation of macrophage function by epigenetics and cytokines. Epigenetics plays a significant role in regulating the inflammatory response and lipid metabolism of macrophages,^529^ and currently focusing on microRNAs, such as miR-21, miR-155, miR-33 and miR-144-3p. In the advanced stage of AS, local delivery of miR-21 to carotid plaque or extensive inhibition of miR-155 expression can inhibit the secretion of inflammatory mediators such as TNF-α, MCP1, IL-6 and IL-1β by macrophages, and promote the expression of IL-10;^530–532^ however, anti-miR-33 and anti-miR-144-3p therapies promote ABCA1 and ABCG1 mediated cholesterol efflux in macrophages, alleviating lipid accumulation and inflammatory response.^533,534^ Based on this, AS plaque burden can be reduced, plaque rupture can be prevented, and the progression of AS can be delayed.^531–534^ It is worth noting that miR-21 can also regulate the function of macrophages in the heart, and the delivery of miR-21 significantly inhibits the macrophage-mediated inflammatory response in the infarcted myocardium, effectively reducing the infarct size and myocardial fibrosis.^535,536^ For cytokines, cytokines such as IL-1, IL-19, and IL-13 play an important role in AS lesions. The administration of IL-19 and IL-13 can activate pathways such as STAT3, STAT6, and KLF4 to promote macrophages to perform anti-inflammatory, lipid efflux, efferocytosis and other functions, and anti-IL-1β antibody can inhibit IL-1-mediated chronic inflammation and lipid metabolism disorders, thereby improving the stability of atherosclerotic plaques.^363,537–539^

In addition, many antihyperglycemic and lipid-lowering drugs that have been clinically applied have also been found to regulate macrophage function, such as Dapagliflozin, Pioglitazone, Sitagliptin and Rosuvastatin. Dapagliflozin is a highly potent and selective sodium-glucose co-transporter 2 (SGLT2) inhibitor that has been shown to reduce fibrosis and AS formation. In terms of regulating fibrosis, Dapagliflozin effectively alleviates myocardial fibrosis after MI by inhibiting macrophage inflammatory pathways (especially NF-κB) and promoting repair function mediated by the RONS/STAT3 pathway.^540,541^ Besides, it can also promote the transformation of M1 macrophages into M2 phenotype by inhibiting LPS-induced TLR-4 overexpression and NF-κB activation in macrophages, reducing the rate of atherosclerotic plaque formation and increasing plaque stability.^542,543^ Pioglitazone is a PPARγ agonist, and intravenous administration after MI reduces infarct and border zone fibrosis by skewing macrophages toward a pro-healing M2 phenotype through inhibition of NF-κB.^544^ Sitagliptin promotes the deflection of macrophages toward the M2 phenotype through SDF-1/CXCR1 signaling, and Rosuvastatin promotes cholesterol efflux and the secretion of anti-inflammatory mediators by increasing the expression of ABCA1, ABCG1, Arg-1 and CD206 in macrophages, so they can reduce the formation of early lesions, alleviate plaque load and prevent further development of AS.^545,546^ Notably, most current studies on the regulation of macrophage function in CVD lack comparisons between the results of intervention at different time points, so it is necessary to strengthen the exploration of the optimal time window for intervention (Table 3).

**Table 3:** Selected published articles related to the current status of preclinical macrophage targeting strategies

Study	Model	Therapeutic strategy	Therapeutic outcome	Citation
**Inhibition of macrophage recruitment**				
Ostermann et al. (2005)	AS	JAM-A inhibition	Soluble JAM-A inhibits JAM-A mediated recruitment of monocytes on atherosclerotic endothelium and reduces inflammation, thereby reducing the formation of atherosclerosis.	^486^
Kentischer et al. (2006)	AS	Anti-MIF monoclonal antibody treatment	MIF blockade strongly reduces macrophage content in the lesions and leads to markedly decreased levels of circulating and local aortic inflammatory mediators, thereby reducing the formation of atherosclerosis.	^489^
Christophe et al. (2008)	AS	Combined inhibition of CCL2, CX3CR1, and CCR5	Combined inhibition of CCL2, CX3CR1, and CCR5 pathways almost abrogates macrophage accumulation and atherosclerosis in mice.	^388^
Wang et al. (2018)	MI	Anti-CCR2 antibody treatment	Inhibiting CCR2 significantly reduces monocyte recruitment in the heart, alleviates inflammatory cascade reactions, and reduces myocardial infarction area.	^601^
Samuel et al. (2023)	AS	VCAM-1 Inhibition	RAG8 treatment reduces VCAM-1 protein levels and platelet accumulation in atherosclerotic coronary arteries, thereby reducing coronary artery atherosclerosis and myocardial fibrosis.	^484^
**Inhibition of foam cell formation and macrophage survival**				
Andrew et al. (2004)	AS	Inducing ABCA1 expression	PPARα and PPARγ agonist therapy induces LXRα and LXR mediated ABCA1 expression which plays a role in promoting cholesterol efflux and reducing the formation of foam cells, ultimately inhibiting the development of atherosclerosis.	^497^
Secchiero et al. (2006)	AS	TRAIL injection	TRAIL injection not only significantly attenuates the total extension of the plaques, but also contributes to stabilize atherosclerotic plaques by selectively decreasing the number of infiltrating macrophages in the atherosclerotic lesions.	^509^
Verheye et al. (2007)	AS	Delivery of everolimus	Stent-based delivery of everolimus selectively clears macrophages in rabbit atherosclerotic plaques by autophagy, thereby reduceing atherosclerosis.	^513^
Petri et al. (2010)	AS	Silence of SR-A	Silencing of SR-A significantly reduces the formation of foam cells, thereby reducing atherosclerosis in mice.	^310^
Duewell et al. (2010)	AS	NLRP3-deficient	The absence of NLRP3 inflammasome significantly protects atherosclerotic mice from disease invasion and reduces the size of lesions.	^602^
Wang et al. (2013)	AS	Downregulation of mTOR expression	The down-regulation of mTOR induces autophagy of macrophages, leading to a decrease in their number and stabilizing atherosclerotic plaque.	^514^
Zhai et al. (2014)	AS	Inhibition of PI3K/Akt/mTOR signaling pathway	Selective inhibition of Akt/mTOR signaling pathway reduces macrophages by promoting autophagy, thereby stabilizing vulnerable atherosclerotic plaque.	^515^
Karunakaran et al. (2016)	AS	Nec-1 treatment	Nec-1 reduces lesion size and markers of plaque instability, including necrotic core formation.	^510^
Shoulders et al. (2019)	AS	Clo-Lip administration	Clo-Lip administration leads to macrophage apoptosis by inhibiting mitochondrial oxygen consumption, thus preventing the progression of atherosclerosis.	^508^
Xu et al. (2023)	AS	IL-37 treatment	IL-37 inhibits iron death of macrophages by activating the NRF2 pathway, thereby slowing down the progression of atherosclerosis.	^512^
Luo et al. (2024)	AS	MCL treatment	MCL activates the NRF2 pathway, thereby inhibiting ferroptosis of macrophages and alleviating the progression of atherosclerosis.	^511^
**Regulation of macrophage function**				
Cardilo-Reis et al. (2012)	AS	IL-13 treatment	IL-13 promotes the production of repair macrophages, thereby stabilizing AS plaques and preventing the development of AS.	^363^
Sager et al. (2015)	MI	Anti-IL-1β treatment	Anti-IL-1β reduces leukocyte infiltration, reduces inflammation in the infarct area, weakens fibrosis, and prevents adverse cardiac remodeling.	^519^
Wei et al. (2015)	AS	MiR-155 inhibition	MiR-155 inhibition promotes macrophage efferocytosis, thereby inhibiting the formation of necrotic core and the progression of atherosclerosis.	^532^
Brenner et al. (2015)	AS	Sitagliptin treatment	Sitagliptin promotes the differentiation of monocytes into the M2 phenotype, reduces plaque burden, and thereby inhibiting early atherosclerosis.	^545^
Gabunia et al. (2016)	AS	IL-19 treatment	IL-19 inhibits macrophage inflammation, maintains cholesterol homeostasis, thereby preventing AS plaque progression.	^537^
Jung et al. (2017)	MI	IL-10 treatment	Infusion of IL-10 at the appropriate period can inhibit post-MI inflammation and reduce collagen deposition by stimulating the polarization of M2 macrophages.	^114^
Price et al. (2017)	AS	MiR-33 inhibition	Anti-miR-33 therapy reduces lipid accumulation and inflammatory responses in macrophages, thereby mediating AS protection.	^533^
Lee et al. (2017)	MI	Dapagliflozin treatment	Dapagliflozin increases the activation of M2 macrophages, thereby inhibiting the differentiation of myofibroblasts and reducing collagen fiber production and alleviating myocardial fibrosis.	^540^
Han et al. (2018)	MI	IL-4pDNA treatment	IL-4pDNA delivery promotes M2 polarization, which reduces cardiac inflammation, weakens fibrosis, and improves cardiac function.	^521^
Jin et al. (2018)	AS	MiR-21 treatment	MiR-21 inhibits the transformation of macrophages into foam cells and relieves the restriction of smooth muscle cells proliferation by activated macrophages, which results in thickening of the fibrous cap and stabilization of AS plaques.	^531^
Podaru et al. (2019)	MI	M-CSF and IL-4-induced macrophage transplantation	Cardiac microvascular formation is enhanced, cardiomyocyte hypertrophy is reduced, and pathological interstitial fibrosis distal to the infarcted area is attenuated.	^527^
Tokutome et al. (2019)	MI	Pioglitazone treatment	Pioglitazone increases M2 macrophage activation, reduces cardiac inflammatory response, and promotes appropriate collagen fiber production.	^544^
Liao et al. (2020)	MI	Heart-derived MSCs infusion	MSCs infusion inhibits macrophage infiltration and induces the development of macrophages toward an anti-inflammatory M2 phenotype, significantly reducing infarct size after AMI and mediating appropriate fibrogenesis in the injured area.	^526^
Zhang et al. (2021)	AS	Rosuvastatin treatment	Rosuvastatin improves macrophage autophagy activity and lipid accumulation, thereby exerting anti-atherosclerotic effects.	^546^
Zhu et al. (2022)	MI	Hypoxia-induced macrophage transplantation	Myocardial cell apoptosis is reduced, angiogenesis is induced, and fibrosis in the infarct area and border zone is attenuated.	^528^
Abdollahi et al. (2022)	AS	Dapagliflozin treatment	Dapagliflozin can inhibit the inflammatory response of macrophages, thereby preventing the progression of AS.	^542^
Chen et al. (2023)	MI	IL-4pDNA treatment	IL-4pDNA promotes M2 polarization, reduces cardiac inflammation, promotes cardiac angiogenesis, and alleviates myocardial fibrosis.	^523^
Wang et al. (2023)	MI	IL-10 treatment	IL-10 delivery promotes M2 polarization, reduces cardiac inflammation, and effectively reduces myocardial fibrosis in the infarct area.	^522^

*AS* atherosclerosis, *CCL2 C-C* motif chemokine ligand 2, *CX3CR1* C-X3-C motif chemokine receptor 1, *CCR* C-C motif chemokine receptor, *VCAM-1* vascular cell adhesion molecule 1, *JAM-A* junctional adhesion molecule A, *MIF* migration inhibitory factor, *MI* myocardial infarction, *SR-A* scavenger receptor A, *ABCA1* ATP-binding cassette transporter A1, *PPAR* peroxisome proliferator-activated receptor, *LXR* liver X receptor, *Clo-Lip* clophosphonate-liposome, *TRAIL* tumor necrosis factor-related apoptosis-inducing ligand, *Nec-1* necrostatin-1, *NLRP3* NOD-like receptor thermal protein domain associated protein 3, *MCL* micheliolide, *NRF2* nuclear factor erythroid related factor 2, *IL* interleukin, *mTOR* mammalian target of rapamycin, *PI3K* phosphatidylinositol-3-kinase, *MSCs* mesenchymal stem cells, *M-CSF* macrophage colony-stimulating factor, *miR* microRNA

## Clinical progress and translational implications

Most current clinical trials on CVD involve anti-inflammatory therapies based on inflammatory cytokines and chemokines (such as IL-1, IL-6, TNF-α, and CCL2) and anti-lipid therapies that inhibit foam cell formation (Table 4). The CANTOS trial is the first successful immunotherapy trial in CVD. A neutralizing antibody canakinumab against IL-1β, an inflammatory cytokine primarily produced by macrophages, was the first drug shown in a clinical trial to specifically and successfully reduce inflammation and the recurrence rate of cardiovascular events in patients after MI.^547^ Colchicine, a broadly anti-inflammatory drug, can not only inhibit the production of IL-1β activated by the NLRP3 inflammasome in macrophages^548^ but also interfere with the TNF-α-induced NF-κΒ pathway^549^ to reduce inflammation. The COLCOT (Colchicine Cardiovascular Outcomes Trial) demonstrated that colchicine treatment in patients with MI significantly reduced the risk of ischemic cardiovascular events such as resuscitative cardiac arrest, MI, stroke, and angina.^550^ The LoDoCo2 (Low-Dose Colchicine for Secondary Prevention of Cardiovascular Disease) trial also demonstrated that 0.5 mg of colchicine once a day significantly reduced the risk of cardiovascular events in patients with chronic coronary artery disease.^551^ As a downstream inflammatory signaling of IL-1, IL-6 also participates in the inflammatory response and immunomodulation, thereby affecting the occurrence and development of CVD.^552^ The ASSAIL-MI trial showed that early treatment with tocilizumab, an IL-6 antibody, enhanced myocardial salvage in patients with STEMI, and there was a tendency to reduce the size of MI.^553^ For anti-lipid therapies, systemic ACAT inhibition has been shown to reduce circulating TNF-α levels and improve vascular endothelial function in hypercholesterolemic subjects.^554^ However, several subsequent trials in patients with hypercholesterolemia^555^ and coronary AS^556,557^ showed that ACAT inhibition did not improve the disease but actually promoted AS. This may be attributed to the fact that inhibition of ACAT-1 causes free cholesterol to accumulate to toxic levels in macrophages, leading to cell death.^555^ Therefore, targeting ACAT as a therapeutic strategy for CVD may need to be considered carefully. Finally, two phase II clinical trials targeting the inhibition of the CCL2-CCR2 axis (NCT01269242, NCT00715169) have been successfully conducted. Treatment with bindarit, a CCL2 inhibitor, could prevent restenosis in patients after percutaneous coronary intervention.^558^ Furthermore, in patients with CVD risk factors, treatment with the anti-CCR2 monoclonal antibody MLN1202 significantly reduced the level of C-reactive protein (CRP), an established biomarker of inflammation associated with coronary artery disease.^268^

**Table 4:** Clinical trials with macrophage-based cardiovascular disease therapeutics

Study	Agent	Drug target	Patient cohort	Enrollment	Primary end point	Main outcomes	Phase	NCT number	Citation
**Inhibition of macrophage recruitment**									
Colombo et al. (2016)	Bindarit	A CCL2 inhibitor	Patients submitted to coronary stenting and using a bare metal stent	148	In-segment late loss	Bindarit helped patients prevent restenosis.	Phase 2	NCT01269242	^558^
Gilbert et al. (2011)	MLN1202	Monoclonal antibody against CCR2	Patients with risk factors for cardiovascular disease	108	The change in median CRP level from baseline to day 57	Patients had significantly lower levels of CRP than the placebo.	Phase 2	NCT00715169	^268^
**Inhibition of foam cell formation and macrophage survival**									
NA	MEDI6570	Antibody against LOX1 receptor	Patients with previous MI	423	Non-calcified plaque volume measured by CTA	NA	Phase 2	NCT04610892	NA
Nissen et al. (2006)	Pactimibe	ACAT inhibitors	Patients with coronary disease	534	NA	Treatment with ACAT inhibitors did not improve percent atheroma volume.	Phase 2	NCT00185042	^556^
Meuwese et al. (2009)	Pactimibe	ACAT inhibitors	Patients with familial hypercholesterolemia	796	The maximum CIMT	Pactimibe had no effect on atherosclerosis but was associated with an increased incidence of major cardiovascular events compared with the placebo.	Phase 2&3	NCT00151788	^555^
Pradhan et al. (2022)	Pemafibrate	A selective PPARα modulator	Patients with diabetes	10,544	A composite of nonfatal MI, ischemic stroke, coronary revascularization, or death from cardiovascular causes	The incidence of cardiovascular events was not lower among those who received pemafibrate than the placebo.	Phase 3	NCT03071692	^603^
Puato et al. (2010)	Atorvastatin	A macrophage accumulation inhibitor	Patients with hypercholesterolemic	60	NA	Macrophage accumulation was significantly reduced in the plaques of patients treated with statins.	NA	NCT01053065	^604^
Elkhawad et al. (2012)	Losmapimod	A p38 MAPK inhibitor	Patients with atherosclerosis on stable statin therapy	99	Change from baseline in average TBR across all segments in the index vessel	High-dose losmapimod reduced vascular inflammation in the most inflamed regions, concurrent with a reduction in inflammatory biomarkers and FDG uptake in visceral fat.	Phase 2	NCT00633022	^605^
O’Donoghue et al. (2016)	Losmapimod	A p38 MAPK inhibitor	Patients with AMI	3503	The composite of cardiovascular death, MI, or severe recurrent ischemia requiring urgent coronary revascularization with the principal analysis specified at week 12	The use of losmapimod compared with placebo did not reduce the risk of major ischemic cardiovascular events.	Phase 3	NCT02145468	^606^
Newby et al. (2014)	Losmapimod	A p38 MAPK inhibitor	Patients with NSTEMI	526	Inflammation (hsCRP concentration at 12 weeks) and infarct size (AUC for troponin I over 72 h or hospital discharge, whichever was earlier)	The p38 MAPK inhibition with losmapimod was well tolerated in NSTEMI patients and might improve outcomes after ACS.	Phase 2	NCT00910962	^607^
Fox et al. (2014)	Ivabradine	A regulatory molecule of PI3K/Akt/mTOR	Patients with stable coronary artery disease	19,102	A composite of death from cardiovascular causes or nonfatal MI	The addition of ivabradine did not improve outcomes.	Phase 3	NCT02446990	^608^
Rodriguez et al. (2012)	Rapamycin	A mTOR inhibitor	Patients with bare metal stent implantation	200	Compare overall costs (in-hospital and follow-up costs of the two revascularization strategies (OR and DES) at 1, 3 and 5 years follow-up	There were no differences in effectiveness between the two revascularization strategies.	Phase 4	NCT00552669	^609^
Stähli et al. (2022)	Everolimus	A mTOR inhibitor	Patients with STEMI undergoing PCI	150	The change in MI size	The inhibition of mTOR with everolimus did not reduce MI size or MVO at 30 days.	Phase 1&2	NCT01529554	^610^
Jamialahmadi et al. (2022)	Trehalose	A macrophage autophagy activator	Patients with history of MI and evidence of systemic inflammation	15	The change in arterial wall inflammation, assessed by quantifying 18F-FDG uptake in carotid arteries and ascending aorta.	No significant reduction in arterial wall inflammation could be observed.	Phase 2	NCT03700424	^611^
**Regulation of macrophage function**									
Tardif et al. (2019)	Colchicine	Broad immunosuppression	Patients with MI within 30 days before enrollment	4745	A composite of death from cardiovascular causes, resuscitated cardiac arrest, MI, stroke, or urgent hospitalization for angina leading to coronary revascularization	Colchicine decreased the risk of the composite endpoint compared with placebo.	Phase 3	NCT02551094	^550^
Nidorf et al. (2020)	Colchicine	Broad immunosuppression	Patients with chronic coronary artery disease	5522	A composite of cardiovascular death, spontaneous MI, ischemic stroke, or ischemia-driven coronary revascularization	Colchicine decreased the risk of the composite endpoint compared with placebo.	Phase 3	ACTRN12614000093684	^551^
NA	Colchicine	Broad immunosuppression	Patients with ACS	500	NA	NA	Phase 4	NCT01906749	NA
NA	Colchicine	Broad immunosuppression	Patients with ACS	80	NA	NA	Phase 2&3	NCT00754819	NA
NA	Colchicine	Broad immunosuppression	Patients with CAD undergoing PCI	132	NA	NA	Phase 4	NCT05130892	NA
NA	Colchicine	Broad immunosuppression	Patients with MI	7063	MACE	NA	Phase 3	NCT03048825	NA
NA	Colchicine	Broad immunosuppression	Patients with ischemic stroke or at high risk of transient ischemic attack	3154	Recurrence of non-fatal ischemic stroke or non-fatal MACE, or vascular-related death	NA	Phase 3	NCT02898610	NA
NA	Hydroxychloroquine	Broad immunosuppression	Patients with MI	125	Rate of cardiovascular adverse events (MI, death, hospitalization for unstable angina and heart failure)	NA	Phase 4	NCT02648464	NA
NA	Hydroxychloroquine	Broad immunosuppression	Patients with CAD and hsCRP >1 mg/l	35	Change in fasting hsCRP level	NA	Phase 4	NCT02874287	NA
Ridker et al. (2019)	Methotrexate	Broad immunosuppression	Patients with atherosclerosis	4786	a composite of nonfatal MI, nonfatal stroke, or cardiovascular death	Low-dose methotrexate did not reduce levels of IL-1β, IL-6, or CRP and did not result in fewer cardiovascular events than placebo.	Phase 3	NCT01594333	^612^
NA	Methotrexate	Broad immunosuppression	Patients with multivessel CAD and hsCRP >2 mg/l	40	Reduction in plaque volume, measured by CTA	NA	Phase 2&3	NCT04616872	NA
Razavi et al. (2018)	Dexamethasone	Broad anti-inflammatory effect	Patients with symptomatic PAD receiving PTA or atherectomy	285	12-month primary patency was defined as a composite of freedom from binary restenosis and clinically driven target lesion revascularization	After 12 months of follow-up, the patient’s restenosis decreased.	Phase 4	NCT01983449	^613^
Ridker et al. (2017)	Canakinumab	Inhibition of the IL-1β pathway	Patients with previous MI and elevated plasma CRP levels	10,066	Nonfatal MI, nonfatal stroke, or cardiovascular death.	The inhibition of the IL-1β pathway with canakinumab led to a significantly lower rate of recurrent cardiovascular events compared with placebo.	Phase 3	NCT01327846	^547^
Abbate et al. (2020)	Anakinra	IL-1 receptor antagonist	Patients with STEMI	99	The AUC for hsCRP, measured at baseline, 72 h, and day 14	The IL-1 blockade with anakinra significantly reduced the systemic inflammatory response compared with placebo.	Phase 2&3	NCT01950299	^614^
Kron et al. (2021)	Anakinra	IL-1 receptor antagonist	Patients with cardiac sarcoidosis	28	Limited to 28 days and additional assessments are for safety purposes only	Patients had significantly lower cardiac and systemic inflammation compared with placebo.	Phase 2	NCT04017936	^615^
Sayed et al. (2016)	Xilonix	Monoclonal antibody specifically targeting IL-1α	Patients undergoing percutaneous SFA revascularization	43	Clinically significant target vessel restenosis, time to restenosis, and incidence of MACE	At 12 months of follow-up, there was no difference between Xilonix and placebo.	Phase 2	NCT01270945	^616^
Ridker et al. (2021)	Ziltivekimab	Monoclonal antibody against IL-6	Patients with chronic kidney disease and hsCRP >2 mg/l	264	hsCRP measured 12 weeks after treatment initiation	Ziltivekimab markedly reduced biomarkers of inflammation and thrombosis relevant to atherosclerosis.	Phase 2	NCT03926117	^617^
NA	Ziltivekimab	Monoclonal antibody against IL-6	Patients with chronic kidney disease and CRP ≥ 2 mg/l	6200	Time to first MACE	NA	Phase 3	NCT05021835	NA
Broch et al. (2021)	Tocilizumab	Monoclonal antibody against IL-6 receptor	Patients within 6 h of STEMI and undergoing PCI	200	The myocardial salvage index as measured by magnetic resonance imaging after 3 to 7 days.	Tocilizumab increased myocardial salvage in patients with acute STEMI.	Phase 2	NCT03004703	^553^
Kleveland et al. (2016)	Tocilizumab	Monoclonal antibody against IL-6 receptor	Patients with NSTEMI	120	The between-group difference in the AUC for hsCRP during hospitalization (days 1–3)	Tocilizumab reduced hsCRP levels compared with the placebo.	Phase 2	NCT01491074	^618^
Meyer et al. (2021)	Tocilizumab	Monoclonal antibody against IL-6 receptor	Patients with out-of-hospital cardiac arrest	80	The reduction in CRP response from baseline until 72 h in patients treated with tocilizumab evaluated by mixed-model analysis for a treatment-by-time interaction	Treatment with tocilizumab resulted in a significant reduction in systemic inflammation and myocardial injury in patients.	Phase 2	NCT03863015	^619^
NA	Sarilumab	Monoclonal antibody against IL-6 receptor	Patients with active rheumatoid arthritis	20	Changes in carotid atheroma plaque assessed by ultrasonography	NA	Phase 4	NCT04350216	NA
NA	Etanercept	A TNF-α inhibitor	Patient with STEMI	200	NA	NA	Phase 4	NCT01372930	NA

*CCL2* C-C motif chemokine ligand 2, CCR2 C-C motif chemokine receptor 2, *CRP* C-reactive protein, LOX1 lectin-like oxidized low-density lipoprotein receptor-1, *MI* myocardial infarction, *CTA* computed tomography angiography, *ACAT* acyl coenzyme A-cholesterol acyltransferase, *CIMT* carotid intima-media thickness, *PPARα* peroxisome proliferator-activated receptor alpha, *MAPK* mitogen-activated protein kinase, *TBR* target-to-background ratio, *FDG* fluorodeoxyglucose, *AMI* acute myocardial infarction, *NSTEMI* non-ST elevation myocardial infarction, *ACS* acute coronary syndrome, *hsCRP* high-sensitivity C-reactive protein, *AUC* area under the curve, *PI3K* phosphatidylinositol 3-kinase, *Akt/PKB* protein kinase B, *mTOR* mammalian target of rapamycin, *STEMI* ST elevation myocardial infarction, *PCI* percutaneous coronary intervention, *MVO* microvascular obstruction, *CAD* coronary artery disease, *MACE* major adverse cardiovascular events, *IL* Interleukin, *PAD* peripheral artery disease, *PTA* percutaneous transluminal angioplasty, *SFA* superficial femoral artery, *TNF* tumor necrosis factor, *NA* not applicable

For the ongoing clinical trials, anti-inflammatory therapies, including broad immunosuppression and those targeting specific cytokines, are primarily utilized. With regard to broad immunosuppression, colchicine is the most widely used and is being tested in patients with acute coronary syndrome (NCT01906749, NCT00754819), coronary heart disease (NCT05130892), MI (NCT03048825), and high-risk patients with ischemic stroke or transient ischemic attack (NCT02898610). The incidence of major adverse cardiovascular events (MACE), such as MI, death, hospitalization for unstable angina, and HF, is evaluated after treatment. Hydroxychloroquine and methotrexate, originally used as broad anti-inflammatory drugs for rheumatism, have both been found to significantly reduce the risk of CVD in patients with rheumatoid arthritis.^559,560^ There are currently two clinical trials ongoing using hydroxychloroquine in patients with MI (NCT02648464) and coronary heart disease (NCT02874287), with the incidence of MACE as the primary endpoint. Additionally, a clinical trial is underway that uses LDL-like nanoparticles to deliver methotrexate to patients with coronary heart disease (NCT04616872). In terms of targeting cytokines, for IL-6, trials of the anti-IL-6 receptor monoclonal antibody sarilumab in patients with rheumatoid arthritis (NCT04350216) and high CRP levels, and of the anti-IL-6 monoclonal antibody ziltivekimab in patients with chronic kidney disease and high CRP levels (NCT05021835) are ongoing, with the changes in atherosclerotic plaques and the incidence of MACE as the primary endpoints, respectively. Targeting the pro-inflammatory cytokine TNF-α, the inhibitor etanercept is also being clinically tested in patients with acute ST-segment elevation myocardial infarction (STEMI) (NCT01372930). Regarding the use of anti-lipid therapy, there is an ongoing clinical trial of the anti-LOX1 receptor antibody MEDI6570 in patients with previous MI, with noncalcified plaque volume as the primary endpoint (NCT04610892). It is expected that the publication of these clinical trial results will bring new insights into the understanding of CVD treatment.

**Fig. 6:**
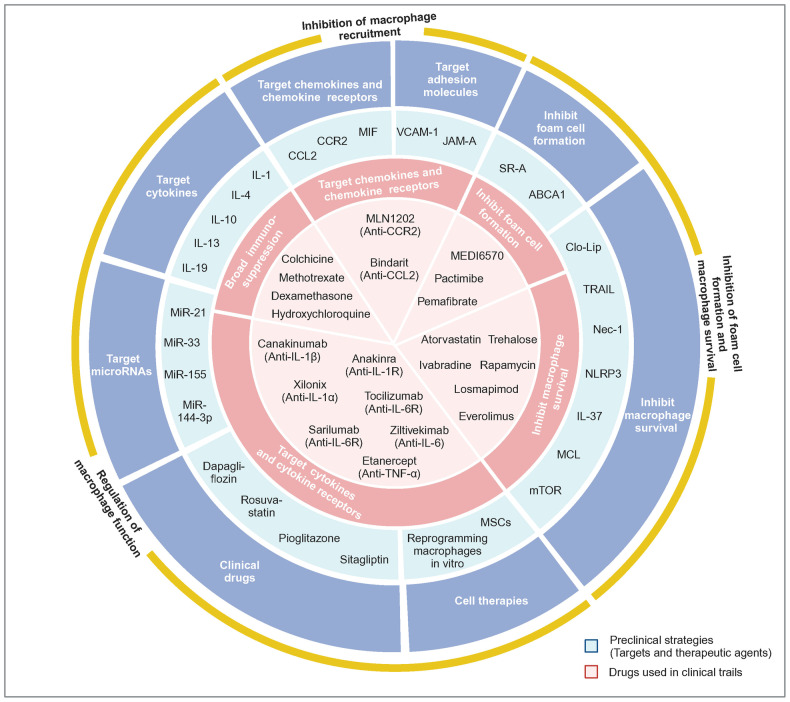
Promising targets for preclinical strategies and clinical trials. This chart outlines promising targets for preclinical strategies and clinical trials aimed at macrophage intervention. These targets primarily focus on three essential mechanisms: inhibition of macrophage recruitment, inhibition of foam cell formation and macrophage survival, and regulation of macrophage function. The blue section underscores targets and therapeutic agents for preclinical strategies on the basis of subdivided macrophage regulatory mechanisms. The red section pertains to drugs currently undergoing clinical trials. (Created with BioRender.com)

Nanomaterials and cell therapy are two promising strategies for the further translation of preclinical treatment modalities for CVD into clinical practice.^475,561–563^ The spatial structures of biomolecules such as cytokines, chemokines, and microRNA are affected by biological, physical and chemical factors such as biological enzymes, temperature, pH, and ionic strength of the surrounding environment in vivo, and also face problems such as off-target and difficulty in breaking through the biofilm barriers, which to a certain extent hinders the efficacy of drugs.^564,565^ Based on the characteristics such as the loading capacity and modifiability,^564,566,567^ nanomaterials can achieve the encapsulation and delivery of biomolecules to isolate the environment in vivo,^568^ assemble themselves with biomolecules or residues to mitigate off-target effects,^536,569,570^ and use material sources with lipid-soluble or positive surface potential properties to help therapeutic drugs cross cell membranes,^571^ which provides a solution to obstacles in the clinical translation of drugs. Cell therapy has the advantages of individualization, durability, and low drug resistance, and can solve refractory CVD that cannot be solved by traditional drugs.^563^ When preparing in vitro reprogrammed macrophages for adoptive transplantation therapy, autologous macrophages are not only less efficient in the collection and processing process, but more importantly, the weak proliferation and difficult genetic manipulation characteristics of macrophages themselves increase the difficulty of modification and expansion in vitro.^506,572^ With induced pluripotent stem cells (iPSCs) from healthy donors as the source, repair macrophages can be prepared in large quantities by utilizing their good plasticity and proliferation, which will greatly improve the efficiency of macrophage-based cell therapy.^572^ MSCs transplantation can contribute to the treatment of CVD, however, MSCs-based cell therapy may cause many adverse reactions in organisms, such as immune response, embolism, graft-versus-host disease, and risk of malignant tumors.^573–576^ The main way for MSCs to exert function is secretion of exosomes, and the infusion of exosomes or further isolation of effector substances in exosomes can minimize safety issues of live cell management, showing reduced immunogenicity and tumor development risk.^577^ It is worth noting that many nanomaterials and cell therapies have been used in various clinical fields, which provides a precedent for clinical translation in the cardiovascular field^563,564^ (Fig. 6).

## Conclusion and perspective

This article comprehensively reviews the regulatory mechanisms of macrophages in ischemic and non-ischemic cardiac injuries, as well as vascular diseases, which involve inflammation responses and their impact on fundamental pathological processes such as myocardial fibrosis, myocardial hypertrophy, myocardial metabolic disorders, and vascular injury. Additionally, the advancements in targeted macrophage therapy have garnered considerable attention in both preclinical strategies and clinical trials. From macrophage recruitment to its role in mediating CVD progression, three characteristics are captured. First, monocyte-derived CCR2^+^ macrophages are considered to be the main macrophage subset that plays a pivotal role in CVD. Multiple factors in different CVD backgrounds can catalyze macrophage recruitment, such as cell death caused by ischemia and viral infection, mechanical stress and neurohumoral systems in PO, ROS in DCM and cardiac aging, the abnormal metabolic state in diabetic cardiomyopathy, endothelial damage and plaque formation in AS, as well as hemodynamic changes in AA.^6^ Second, macrophage-secreted mediators may exhibit diverse roles contingent upon different etiologies or different stages of the same etiology. For example, MMP-9 exerts pro-inflammatory and pro-fibrotic effects in AMI^78,79^ and cardiac aging,^254^ while in viral myocarditis, it mitigates myocardial damage and fibrosis by impeding viral proliferation.^207^ IL-1β is involved in both pro-inflammatory and pro-fibrotic processes in AMI,^70^ IRI,^65^ and viral myocarditis.^208^ Nonetheless, it should be noted that early inhibition of IL-1β in AMI leads to insufficient scar formation and cardiac rupture,^578^ while its early suppression in IRI can diminish infarct size and ameliorate ventricular remodeling.^65^ Third, there may exist some shared pathways that play a crucial role across various diseases, and these crucial pathways may intricately intertwine within the same disease, collectively driving disease progression. For instance, the NLRP3/IL-1 axis lays a solid foundation for initiating inflammation, amplifying inflammation, and promoting myocardial apoptosis in a variety of diseases, such as AMI, PO, and AS. OPN plays a pro-fibrotic role in myocarditis,^210^ diabetic cardiomyopathy,^242^ and especially the MI reparative phase.^109,110^ Galectin-3 exhibits an important pro-fibrotic function in CMI^128,129^ and is also significantly upregulated in AMI,^98,99^ PO,^171^ and diabetic cardiomyopathy.^237^ Therefore, based on the complexity of macrophage-mediated signaling pathways in cardiovascular pathogenesis, simplistic applications of cellular mediators like IL-1, IL-10, and MMP-9 often fail to yield expected benefits, elucidating the challenges encountered in most cardiovascular clinical trials when in pursuit of efficacy.^579,580^ In the future, it is imperative to investigate more critical pro-inflammatory and pro-fibrotic mediators that underlie pathological cardiac remodeling and ensure these molecules do not induce severe adverse reactions. Notably, heart failure with preserved or reduced ejection fraction is an increasingly intriguing topic.^581^ As HF signifies the advanced stage of both ischemic and non-ischemic myocardial injury, the macrophage-mediated pathophysiological mechanisms exhibit a degree of convergence.^23^

In the future, optimizing the following aspects may help to further enhance the conversion and success rates of targeted macrophage therapy to cardiovascular clinical practice, including the refinement of macrophage typing to achieve greater precision and granularity, the exploration of novel research directions, the development of accurate disease models, and the implementation of specific treatment approaches. Advancements in single-cell sequencing offer opportunities for further subdivision of macrophage subsets. Currently, there is a lack of precision in targeting specific macrophage types, with most therapeutic strategies tending to concentrate on promoting the polarization of M2 macrophages and related anti-inflammatory mediators. Such a description may account for the fact that the overall functional shift of macrophages is advantageous for disease recovery. However, certain sub-subtypes of the M2 phenotype are not favorable for disease prognosis, and exclusion of these types of macrophages, such as foam cells in AS, may potentially achieve a better therapeutic effect. Meanwhile, the promising therapeutic value of some newly discovered mechanisms in the treatment of CVD, such as macrophage extracellular traps (MET), warrants further investigation. Although MET has garnered significant interest in fields encompassing pathogen infection, acute kidney injury and cystic fibrosis, its potential role in the cardiovascular field has received limited attention.^582,583^ In light of preclinical tests, the problems existing in animal models are gradually revealed and improved. For instance, while permanent coronary artery ligation is widely used to simulate AMI, clinical patients have universal access to reperfusion therapy instead. Traditional MI models generally entail pericardial destruction to access the coronary arteries, which may interfere with cardiac repair.^584,585^ The necessity for more accurate and precise models is highlighted by the fact that fibrosis typically manifests in rodent models over weeks or months, whereas it takes years or decades to develop in humans. Several methods, including the utilization of organoids, heart-on-a-chip, and humanized mice, have been established to investigate disease mechanisms, elucidate cell-to-cell interactions, and conduct drug screening.^586^ In the meantime, the employment of single-cell resolution analyses is aiding in refining in vivo and in vitro models that recapitulate the phenotypes and functions of immune cells, including macrophages. In the management of CVD, there is a notable absence of exploration into the intervention time window, which may be due to the difficulty in controlling the specific stage of disease progression during the experimental procedures. The lack of exploration of the time window for intervention also makes the delicate balance between pro-and anti-inflammatory cells in vivo elusive. It is widely recognized that managing inflammation early or facilitating M2 macrophage polarization during the transition to an anti-inflammatory environment is beneficial for cardiac remodeling, taking the crossover point between the inflammatory and reparative phases of AMI (e.g., 3–4 days after AMI) and early PO as examples. What’s more, by employing small molecules such as miRNAs and antisense oligonucleotides (ASOs) or novel delivery systems such as nanoparticles and hydrogels, compounds are likely to be more effective and target specific without jeopardizing their critical roles in other physiological functions and avoiding catastrophic side effects, which paves the road for clinical translation of preclinical strategies and immunomodulation of CVD.^587^
